# Detection of litchi fruit maturity states based on unmanned aerial vehicle remote sensing and improved YOLOv8 model

**DOI:** 10.3389/fpls.2025.1568237

**Published:** 2025-04-16

**Authors:** Changjiang Liang, Dandan Liu, Weiyi Ge, Wenzhong Huang, Yubin Lan, Yongbing Long

**Affiliations:** ^1^ College of Electronic Engineering/College of Artificial Intelligence, South China Agricultural University, Guangzhou, China; ^2^ National Center for International Collaboration Research on Precision Agricultural Aviation Pesticides Spraying Technology, Guangzhou, China; ^3^ Guangdong Laboratory of Lingnan Modern Agriculture, Guangzhou, China; ^4^ South China Smart Agriculture Public Research & Development Center, Ministry of Agriculture and Rural Affairs, Guangzhou, China

**Keywords:** UAV, YOLOv8, litchi fruit, maturity state, object detection

## Abstract

Rapid and accurate detection of the maturity state of litchi fruits is crucial for orchard management and picking period prediction. However, existing studies are largely limited to the binary classification of immature and mature fruits, lacking dynamic evaluation and precise prediction of maturity states. To address these limitations, this study proposed a method for detecting litchi maturity states based on UAV remote sensing and YOLOv8-FPDW. The YOLOv8-FPDW model integrated FasterNet, ParNetAttention, DADet, and Wiou modules, achieving a mean average precision (mAP) of 87.7%. The weight, parameter count, and computational load of the model were reduced by 17.5%, 19.0%, and 9.9%, respectively. The improved model demonstrated robust performance in different application scenarios. The proposed target quantity differential strategy effectively reduced the detection error for semi-mature fruits by 12.58%. The results showed significant stage-based changes in the maturity states of litchi fruits: during the rapid growth phase, the fruit count increased by 18.28%; during the maturity differentiation phase, semi-mature fruits accounted for approximately 53%; and during the peak maturity phase, mature fruits exceeded 50%, with a fruit drop rate of 11.46%. In addition, YOLOv8-FPDW was more competitive than mainstream object detection algorithms. The study predicted the optimal harvest period for litchis, providing scientific support for orchard batch harvesting and fine management.

## 1 Introduction

Litchi is an important subtropical economic crop (Liang et al., 2023; Wang et al., 2022b). China is the leading producer of litchi, accounting for approximately half of the global annual production (Xiong et al., 2023; Li et al., 2024). Moreover, litchi is characterized by a high yield, a short harvesting period, concentrated market timing, and challenges related to preservation (Liang et al., 2025). Therefore, accurately assessing the maturity state of litchi fruits and determining the optimal harvesting time is crucial for farmers. Litchis that are harvested too early usually lack sufficient sweetness, which reduces their market competitiveness, while late harvesting may lead to overripe fruits or natural drop, thereby reducing the income of fruit farmers. Thus, precise classification of the maturity state is essential for determining the optimal harvest timing. Traditionally, the maturity state and optimal harvest period of litchi fruits are mainly determined by experienced farmers, who visually assess the skin color of the fruit and the proportion of mature fruits visible on the tree. Once a certain proportion of mature fruits is reached, farmers consider it the right time to harvest. However, this method, which relies on manual experience, is not only time-consuming and labor-intensive, but also subject to subjective bias, making it unsuitable for large-scale orchards. Therefore, it is of great significance to develop accurate and efficient litchi fruit maturity state detection technology.

With the development of agricultural digitization, UAV remote sensing technology has become an essential tool for large-scale orchard management due to its wide coverage, high imaging efficiency, and strong adaptability (Hadas et al., 2019; Modica et al., 2020; Wu et al., 2020; Zhang et al., 2021, 2023). Meanwhile, the rapid development of deep learning technology has shown excellent performance in the field of object detection, and it has been widely applied in fruit detection and classification tasks in orchards (Gao et al., 2020; Gené-Mola et al., 2020). Deep learning-based algorithms are primarily divided into two categories: two-stage algorithms, such as Region-Based Convolutional Neural Network (R-CNN) (Girshick et al., 2014), Fast Region-Based Convolutional Neural Network (Fast R-CNN) (Girshick, 2015), and Faster Region-Based Convolutional Neural Network (Faster R-CNN) (Ren et al., 2017), and one-stage algorithms, such as You Only Look Once (YOLO) (Redmon et al., 2016) and Single Shot MultiBox Detector (SSD) (Liu et al., 2016). However, two-stage object detection algorithms are typically slower and cannot achieve real-time detection (Lin et al., 2022; Ghasemi et al., 2022; Bouguettaya et al., 2022; Wang et al., 2022b). In contrast, one-stage object detection algorithms, represented by the YOLO series, offer real-time detection capabilities and lower computational resource requirements, making them more suitable for practical applications (Lin et al., 2017; Zhao et al., 2019; Maity et al., 2021).

Currently, deep learning technology has been applied to various fruit detection tasks, achieving remarkable results (Apolo-Apolo et al., 2020; Vasconez et al., 2020; Liu et al., 2020; Nan et al., 2023). For litchi fruit detection, some studies developed specialized models based on improved YOLO algorithms. Li et al. (2022) improved the YOLOv3-tiny network architecture and proposed the YOLOv3-tiny-Litchi algorithm for identifying densely distributed litchi fruits in orchard environments, achieving an accuracy of 87.43%. Wu et al. (2022) proposed the Lit-YOLO model based on YOLOv4, achieving an accuracy of 85.45%. Li et al. (2024) proposed an improved YOLOv7-Litchi algorithm by integrating multiple modules, such as CNeB and CBAM, into YOLOv7, achieving a precision of 94.60%. However, these studies primarily relied on ground camera data, which struggled to fully cover the fruits at the top of the tree canopy, and they had limitations in large-scale orchard applications. In contrast, the exploration of UAV-based litchi recognition methods has been limited, with only a few studies reporting relatively low accuracy rates. For example, Xie et al. (2022) used data collected from both ground cameras and UAVs to propose an improved litchi fruit detection model, YOLOv5-litchi, with a mean average precision (mAP) of 87.1%. Xiong et al. (2023) developed the YOLOv5-TinyLitchi model for detecting litchi fruits in UAV remote sensing images, achieving an average accuracy of 72.6% by optimizing the loss function and integrating the BiFPN and SAHI modules.

In the field of fruit maturity detection, deep learning techniques have made significant progress in research on fruits such as apples, strawberries, and blueberries. Yang et al. (2023) proposed a strawberry maturity detection and grading model, LS-YOLOv8s, which improved the accuracy by 0.5% compared to the original YOLOv8s. Chen et al. (2024) introduced a multi-task deep convolutional neural network (MTD-YOLO) for grading the maturity stages of tomato fruits, achieving a detection accuracy of 86.6%. Wang et al. (2022a) proposed a detail semantic enhancement model, DSE-YOLO, for multi-stage strawberry fruit detection, achieving a mAP value of 86.58%. Tian et al. (2019) used YOLOv3 to detect three maturity stages of apples, improving the F1 score from 0.793 to 0.817 by adjusting network resolution and adding CNN layers. Liu et al. (2023) added a MobileNetv3 network fused with CBAM, proposing a YOLOv5-based blueberry ripeness detection algorithm, achieving a mAP of 78.3%. However, research on litchi fruit maturity detection has been relatively limited. Wang et al. (2022b) replaced the backbone network of YOLOv5 with ShuffleNetv2 and integrated the CBAM attention mechanism to perform two-class detection of litchi fruits (immature and mature), achieving an accuracy of 92.4%. Similarly, Xiong et al. (2023) developed the YOLOv5-TinyLitchi model, focusing on two-class detection of litchi fruits, achieving an accuracy of 72.6%. However, these studies were limited to simple maturity classification and overlook the transition phase of semi-mature fruits, making it difficult to achieve a fine-grained evaluation of the dynamic maturity of fruits. Additionally, this classification approach had limited capability for dynamically monitoring fruit maturity in large-scale orchards, making it difficult to meet the needs of modern orchard management.

To address the above issues, this study proposed a litchi maturity state detection method based on UAV remote sensing technology and the improved YOLOv8 model (YOLOv8-FPDW). By integrating FasterNet, ParNetAttention, DADet, and Wiou modules, YOLOv8-FPDW achieved accurate classification and detection of litchi fruits at different maturity states (immature fruits, semi-mature fruits, and mature fruits). Additionally, the incorporation of a target quantity differential strategy further enhanced detection accuracy and the ability to assess the growth dynamics of the fruits. This study provides technical support for harvesting planning and precision management in litchi orchards.

## 2 Materials and methods

### 2.1 Data collection

**Figure 1:**
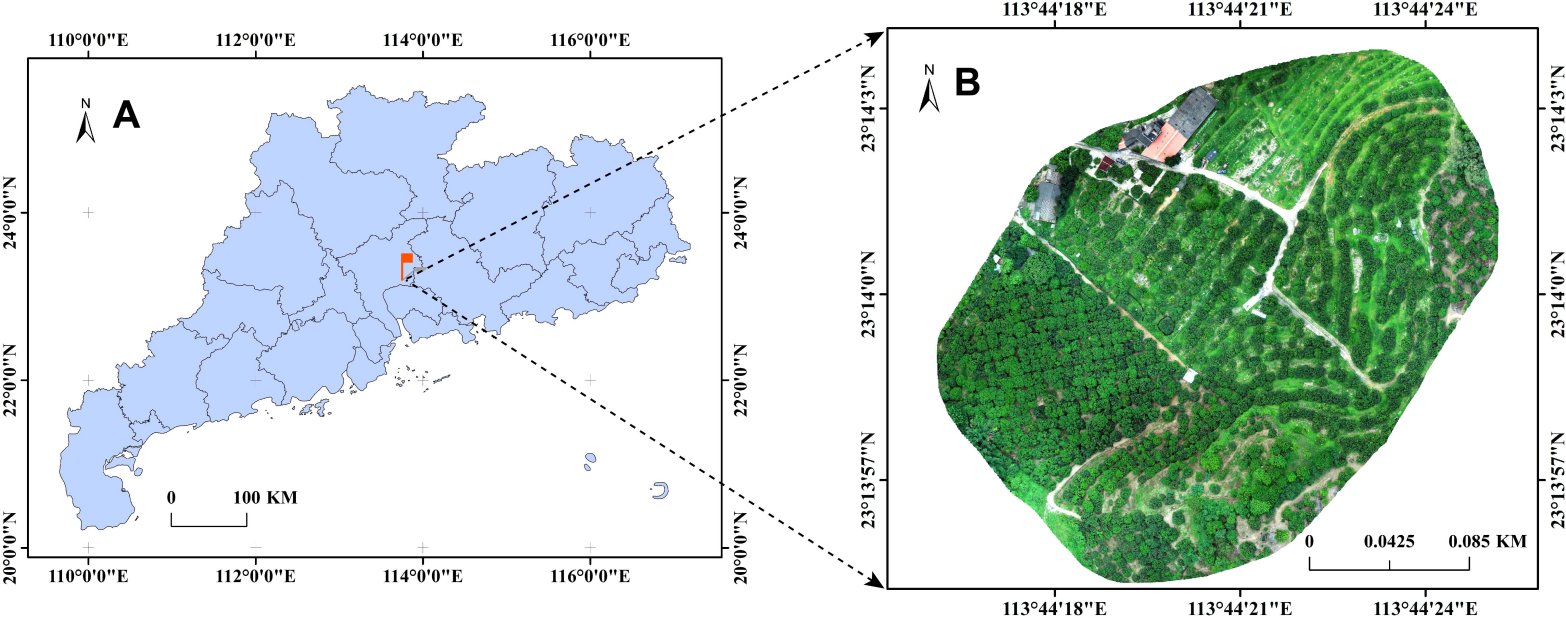
Location of data collection. **(A)** Detailed map of Guangdong Province. **(B)** Aerial view of Sanzhen Fruit Orchard.

The remote sensing images used in this study were collected from the Sanzhen orchard in Zengcheng, Guangzhou (23°14’1.39’’N - 113°44’21.14’’E) (see **Figure 1**), where the primary varieties grown are Xianjinfeng and Guiwei. The data collection spanned from May 20, 2023 (Node 1), June 1, 2023 (Node 2), June 14, 2023 (Node 3), to June 19, 2023 (Node 4), corresponding to different growth stages of the litchi. The remote sensing images were captured by a PHANTOM 4 RTK drone (DJI, Shenzhen, Guangdong) at a height of 6 meters above the tree canopy, with the camera oriented vertically toward the canopy. The resolution of the images was 5472×3648 pixels (aspect ratio of 1.5), and the automatic exposure mode was employed. The ground sampling distance (GSD) ranged from 0.19 to 0.25 cm/pixel. Image capture took place between 9:30 AM and 5:30 PM, resulting in a total of 608 remote sensing images.

### 2.2 Image annotation and dataset production

**Figure 2:**
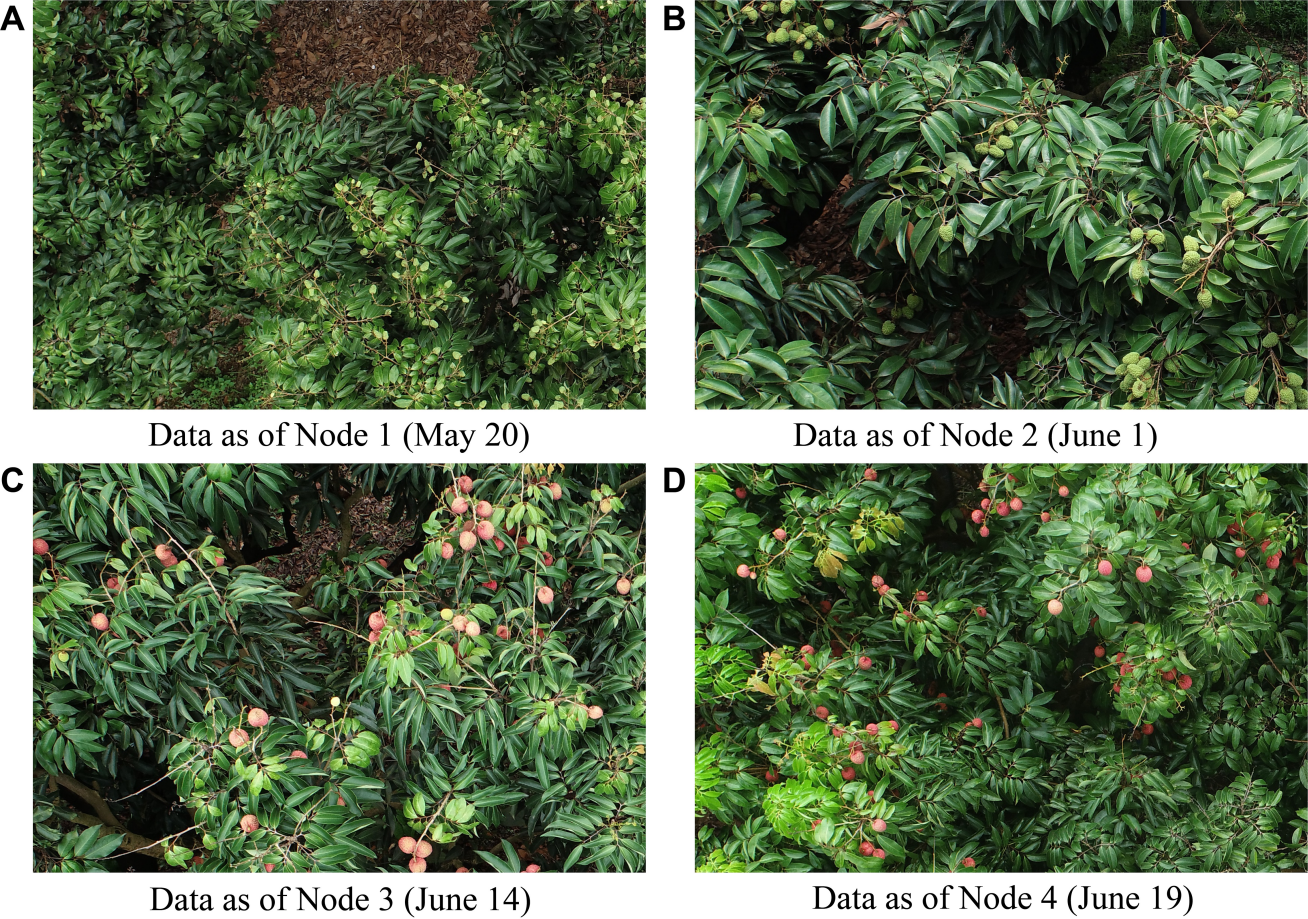
Remote sensing images of litchi fruits at different growth stages. **(A)** Data as of Node 1 (May 20). **(B)** Data as of Node 2 (June 1). **(C)** Data as of Node 3 (June 14). **(D)** Data as of Node 4 (June 19).

The collected remote sensing images exhibited distinct morphological and color differences in litchi fruits at various growth stages (see **Figure 2**). To construct a dataset for litchi fruit maturity detection, 100 images were equally randomly selected from each growth stage, resulting in an initial dataset of 400 images. The original image size of 5472×3648 pixels was deemed unsuitable for model training, so the images were cropped into smaller patches of 1800×1200 pixels (aspect ratio 1.5). Background images without litchi fruits were removed, and a final dataset containing 1126 images was created. Based on the color changes of the litchi fruit peel and the extended BBCH-scale (Wei et al., 2013), the maturities of the fruits were categorized into three stages: immature fruits (BBCH 703-709, litchi1), semi-mature fruits (BBCH 800-807, litchi2), and mature fruits (BBCH 808-809, litchi3), as shown in **Supplementary Figure S1** in the **Supplementary Material**. The BBCH-scale provides a standardized system for categorizing the phenological stages of litchi fruits, where stages 703-709 correspond to immature fruits, 800-807 to semi-mature fruits, and 808-809 to mature fruits, based on color changes in the fruit peel and overall fruit development. The images were manually annotated using the Python-based LabelImg software. After annotation, the dataset was randomly divided into a training set (900 images), a validation set (113 images), and a test set (113 images) in a ratio of 8:1:1.

**Figure 3:**
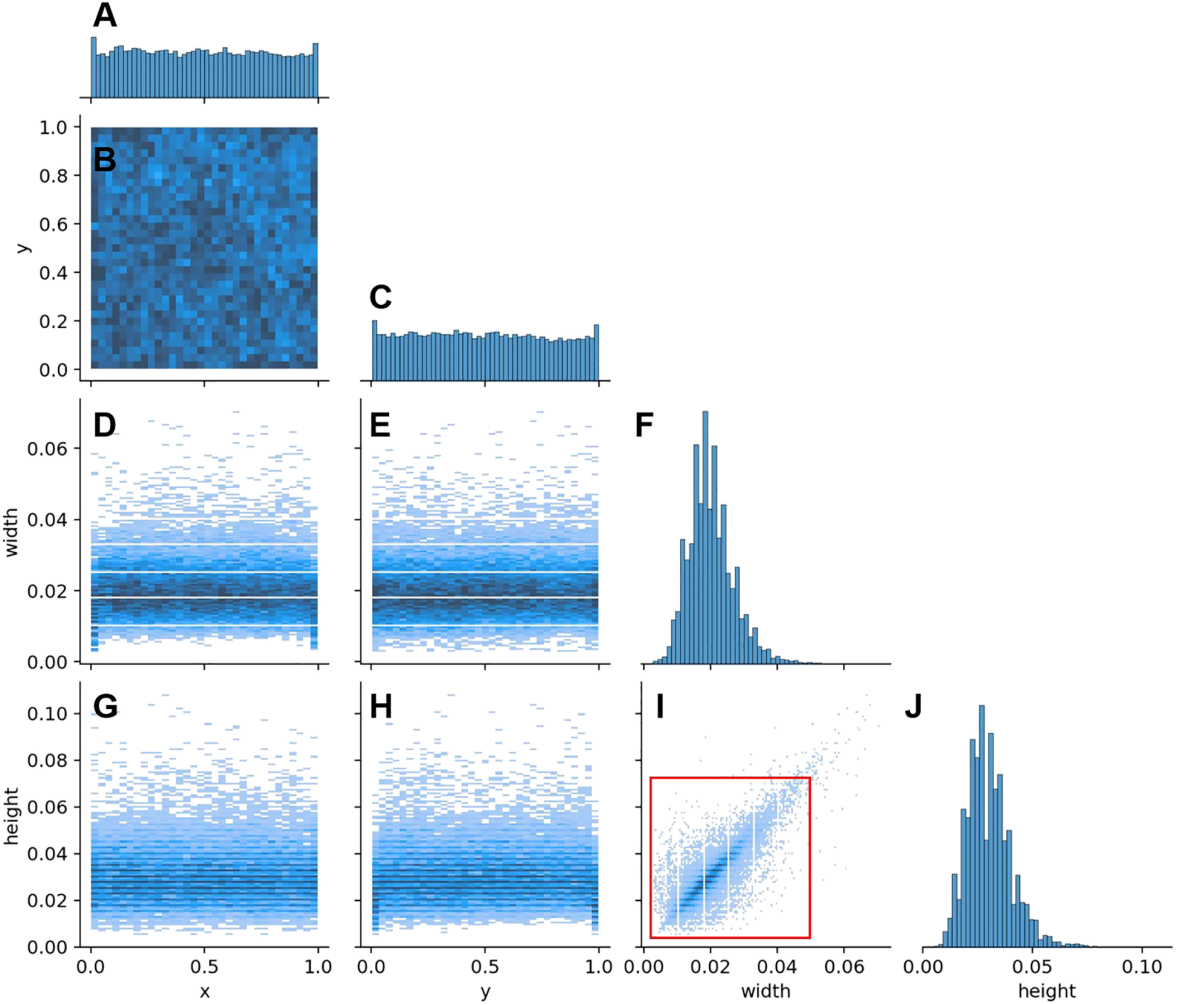
Distribution and dimensions of litchi fruits in the dataset. **(A)** Target distribution frequency along the horizontal axis. **(B)** Distribution of targets in the image, with x and y representing the horizontal and vertical positions of the targets, respectively. **(C)** Target distribution frequency along the vertical axis. **(D)** Relationship between target width and its horizontal position. **(E)** Relationship between target width and its vertical position. **(F)** Histogram of target widths, showing the distribution of target widths in the dataset. **(G)** Relationship between target height and its horizontal position. **(H)** Relationship between target height and its vertical position. **(I)** Relationship between target height and width, with the red rectangle indicating the size range of most targets. **(J)** Histogram of target heights, showing the distribution of target heights in the dataset.

The dataset included a total of 50,703 annotated targets, with 18,655 litchi1 targets, 13,065 litchi2 targets, and 18,983 litchi3 targets. According to the definition standard by Li et al. (2018), a target was classified as a small object when its aspect ratio relative to the entire image was less than 0.1. **Figure 3** displays the size distribution of the targets in the dataset, where most litchi fruits have an aspect ratio smaller than 0.05 relative to the whole image (highlighted by red box in **Figure 3G**). This indicates that small objects constitute a large proportion of our dataset.

### 2.3 Overview of YOLOv8 architecture and key components

YOLOv8 is an improved model in the YOLO object detection series. The network structure of YOLOv8 consists of three parts: the backbone network, the neck network, and the head network, which are responsible for feature extraction, feature fusion, and object detection, respectively. The backbone network optimizes gradient propagation and computational efficiency through the C2f module, thereby enhancing the feature hierarchy representation ability. The neck network uses the PAFPN structure, which enhances the integration of multi-scale information through bottom-up and top-down feature fusion. It also introduces the SPPF module to reduce computational load and improve detection efficiency. The head network adopts an anchor-free mechanism and a decoupled design, with independent classification and regression tasks, optimizing detection accuracy and convergence speed.

### 2.4 Development and architectural design of the YOLOv8-FPDW model

**Figure 4:**
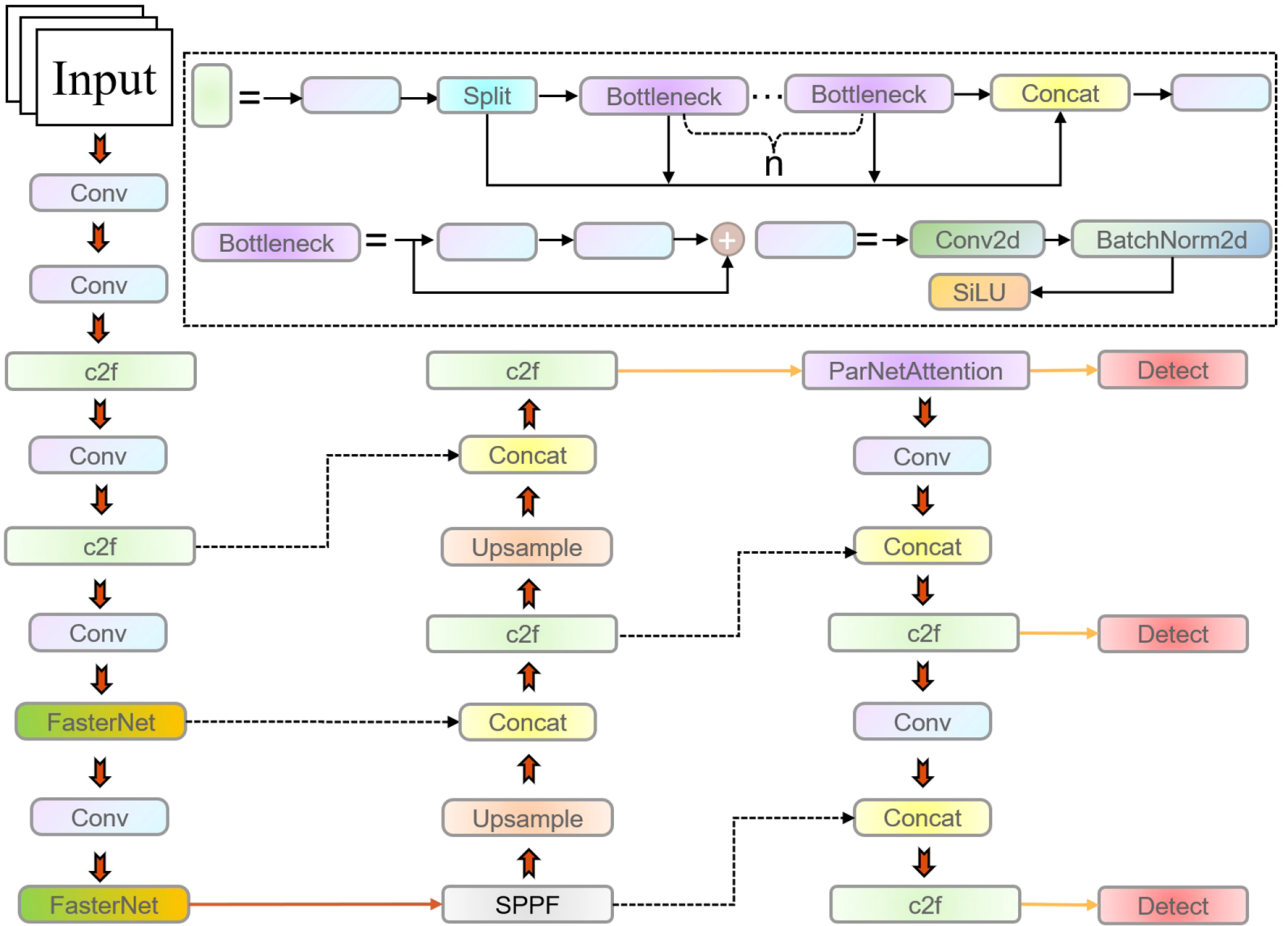
The network structure of YOLOv8-FPDW.

To enhance the detection performance of litchi fruits at various stages of maturity while reducing computational complexity, this study developed an improved version of the YOLOv8 model, termed YOLOv8-FPDW (see **Figure 4**). The model integrated several optimization modules to boost both feature extraction and object detection capabilities. First, the FasterNet module, which combines partial convolution (PConv) and pointwise convolution (PWConv), improves feature extraction efficiency and extends the receptive field. Next, the ParNetAttention mechanism integrates fuses from different resolutions and employs structural reparameterization techniques to enhance model performance. Additionally, the DADet detection head, which combines depthwise separable convolution and the ShuffleNet module, further enhances detection accuracy. Finally, the Wiou loss function is integrated to optimize bounding box regression, improving both localization precision and model generalization. Subsequent sections will provide more detailed implementation aspects and theoretical explanations.

#### 2.4.1 Addition of the FasterNet module

FasterNet (Chen et al., 2023) is a lightweight neural network designed to maintain high speed and low computational cost while enhancing feature extraction capabilities and expanding the receptive field (see **Supplementary Figure S2** in the **Supplementary Material**). The network performs multi-stage feature extraction to assist in the target detection task, with the Faster Block being its core component. The Faster Block employs a combination of PConv and PWConv, structured as an inverted residual block. PConv reduces computation and memory requirements by convolving only a subset of input channels, significantly improving computational efficiency. PWConv, on the other hand, expands the number of channels and utilizes shortcut connections to enhance feature diversity and robustness. This design enables FasterNet to achieve high-quality feature extraction with low computational complexity.

#### 2.4.2 Integration of the ParNetAttention module

ParNetAttention (Goyal et al., 2022) is a lightweight attention mechanism (see **Supplementary Figure S3** in the **Supplementary Material**). This module effectively integrates features of different resolutions through a multi-branch parallel structure. ParNetAttention optimizes feature representations during the training phase using structural reparameterization techniques and reduces computational cost during the inference phase. Furthermore, the downsampling module of ParNetAttention combines Squeeze-and-Excitation (SE) modules with average pooling layers to further compress the number of parameters while retaining the critical information necessary for target detection. This mechanism not only enhances the ability of the model to focus on features in multi-object scenarios but also improves its ability to perform fine-grained detection of target regions.

#### 2.4.3 Design of the DADet detection head

To optimize the classification and localization performance of litchi fruit detection, an improved detection head, DADet (Decoupled-ATSS Detection), was designed (see **Supplementary Figure S4** in the **Supplementary Material**). DADet employs depthwise separable convolutions (DWConv) and the ShuffleNet module, which reduce the number of parameters and computational load while enhancing both classification and bounding box regression performance. Specifically, the detection head splits the feature map into a classification branch and a regression branch. The classification branch focuses on target category identification, using DWConv to reduce computational cost, while the regression branch optimizes bounding box coordinates and improves efficiency with ShuffleNet. Additionally, DADet integrates the ATSS (Adaptive Training Sample Selection) strategy, which adaptively selects positive and negative samples, further improving the detection accuracy of the model.

#### 2.4.4 Addition of the Wiou loss function

Wiou (Tong et al., 2023) is a loss function designed for bounding box regression (BBR), aimed at improving the localization performance of target detection models through a dynamic focusing mechanism. Traditional BBR loss functions often weaken the localization ability of the model when dealing with low-quality samples. In contrast, Wiou utilizes a dynamic non-monotonic focusing mechanism that evaluates anchor box quality based on anomaly scores rather than the IoU value. This allows for more reasonable gradient distribution. This mechanism reduces the negative impact of low-quality samples, while preventing excessive competition among high-quality samples, effectively enhancing the generalization ability and localization accuracy of the model.

### 2.5 Slicing aided hyper inference

SAHI is a slicing-aided inference method designed to address the challenge of detecting small targets in high-resolution images, while also optimizing memory utilization (Akyon et al., 2022). During inference, the original high-resolution image is first divided into multiple overlapping slices (P^I1^, P^I2^,…, P^IL^), with each slice resized while maintaining its aspect ratio. Each slice is then individually input into the target detection model for forward inference to detect small targets. Additionally, the original image is also processed for large target detection. Finally, the results from the slice inference and the original image inference are merged into a unified-scale final detection result using Non-Maximum Suppression (NMS) algorithm. In the NMS phase, only prediction boxes with confidence scores above a predefined matching threshold are retained, and redundant boxes are removed. For further details, refer to **Supplementary Figure S5** in the **Supplementary Material**.

### 2.6 Training environment and evaluation indicators

This study conducted model training and testing under the Ubuntu 18.04.5 operating system, with hardware configurations including 64GB of memory, an NVIDIA GeForce RTX 3090 GPU, and a 64-bit Linux system. Model building and training were implemented using Python3.7.13, Pytorch1.9.1, and CUDA11.1. All models and additional modules in this study were implemented and integrated within this environment. In accordance with the requirements of the YOLOv8 network, images were resized to a resolution of 1280×1280 pixels as input for the model. The training process involved 200 epochs with a batch size of 4, and all other hyperparameters followed the default settings of the official implementation.

The evaluation metrics for this study include mAP, model weight, number of parameters (Parameters), and computational load (GFLOPs). Among these metrics, mAP is the primary metric for assessing target detection accuracy. The calculation formulae are detailed in Equations 1–4. Model weight measures the storage size of the model (in MB). Smaller models are advantageous for deployment in resource-constrained environments (such as mobile devices or UAV platforms). Parameters reflect the complexity of the model, with fewer parameters reducing both storage demands and training costs. GFLOPs (Floating Point Operations Per Second) quantify the computational load, indicating the consumption of computational resources by the model.


(1) $$P=\frac{TP}{TP+FP}$$

(2) $$R=\frac{TP}{TP+FN}$$

(3) $$AP(K)=\int_{0}^{1}P(R)dR$$

(4) $$mAP=\frac{\sum_{1}^{Q}AP(K)}{Q}$$

In the formulae, *TP* represents the number of true positive samples predicted by the model, *FP* represents the number of false positive samples predicted by the model, and *FN* represents the number of false negative samples predicted by the model.

## 3 Experimental results and analysis

### 3.1 Ablation experiment of the improved model

To assess the individual contribution of each additional module in the YOLOv8-FPDW model to the detection performance, we conducted a systematic ablation study on a litchi detection dataset with different maturity states. Using the original YOLOv8 model as the baseline, we progressively integrated modules such as FasterNet, ParNetAttention, DADet, and Wiou into the baseline model, creating multiple improved models. A comprehensive comparison of their performance was conducted, and the experimental results are shown in **Table 1**. The mAP@0.5 of the YOLOv8-FPDW algorithm reached 87.7%, representing an increase of 2.7% over the original YOLOv8 algorithm (85.0%). The detailed functions of each module and their roles in improving the algorithm were further analyzed in the following subsections.

**Table 1:** Performance comparison of original YOLOv8 model and YOLOv8 integrated with various modules.

Model	AP@0.50	AP@0.95	Weight	Parameters	GFLOPs
YOLOv8	85.0	65.4	6.3Mb	3006233	8.1
v8+FasterNet	86.3	67.2	5.6Mb	2636825	7.5
v8+ParNetAttention	85.6	66.3	6.4Mb	3051737	8.6
v8+ DADet	85.6	66.2	5.9Mb	2759801	**7.3**
v8+Wiou	85.8	66.2	6.3Mb	3006233	8.1
YOLOv8-FPDW	**87.7**	**67.6**	**5.2Mb**	**2435897**	**7.3**

Bold values indicate the optimal results in each column.

#### 3.1.1 Contribution of FasterNet module to detection performance

After integrating the FasterNet module into the YOLOv8 model, the mAP@0.5 increased from 85.0% to 86.3% (see **Table 1**). The improvement in detection accuracy was due to the fact that FasterNet enhanced the feature expression capability through efficient feature extraction and expanded receptive field, thereby improving detection performance. Additionally, this module optimized the lightweight design of the model, reducing the weight from 6.3MB to 5.6MB (a decrease of 11.1%), and decreasing the Parameters and GFLOPs by 12.3% and 7.4%, respectively. This lightweight design significantly reduced computational load and storage requirements, making it suitable for resource-limited agricultural scenarios.

#### 3.1.2 Contribution of ParNetAttention to detection performance

After integrating the ParNetAttention module, the mAP@0.5 of the improved model increased by 0.6%, reaching 85.6% (see **Table 1**). This module enhanced the ability of the feature map to extract key information through an attention mechanism, thereby improving the detection accuracy of the model. Although the introduction of ParNetAttention slightly increased the weight of the model (by 1.6%) and parameter count (by 1.5%), and GFLOPs increased from 8.1 to 8.6, the improvement in detection performance indicated that this computational overhead was well worth the trade-off. It also demonstrated that ParNetAttention is a lightweight module, effectively focusing on target areas and suppressing background noise, which improved the performance of model in detecting litchi fruits in remote sensing images.

#### 3.1.3 Combined effect of DADet to detection performance

After integrating the DADet module into the YOLOv8 model, the mAP@0.5 increased from 85.0% to 85.6% (see **Table 1**). This performance improvement was attributed to the more refined decoupling design of classification and regression tasks in the DADet module. Additionally, by incorporating DWConv and ShuffleNet, computational redundancy was reduced, and feature expression efficiency was enhanced. In terms of lightweight design, the DADet module significantly optimized the resource requirements of the model, reducing model weight, parameter count, and GFLOPs by 6.3%, 8.2%, and 9.9%, respectively. This design balanced high detection performance while lowering resource demand, making the model more suitable for resource-constrained and rapid-response agricultural applications.

#### 3.1.4 Contribution of Wiou to detection performance

After introducing the Wiou loss function into the YOLOv8 model, the detection accuracy improved by 0.8% (see **Table 1**). This increase in accuracy was due to the dynamic non-monotonic focus mechanism of the Wiou module, which optimized the anchor box regression process. Unlike the traditional IoU loss function, Wiou dynamically adjusted gradient gain distribution, thereby improving the robustness and localization performance of the model. Moreover, the introduction of Wiou did not increase the model weight, Parameters, or computational complexity, demonstrating that the performance improvement was achieved without consuming additional resources. The design of Wiou made it suitable for fine-grained classification tasks in litchi maturity detection.

#### 3.1.5 Comprehensive performance analysis of the YOLOv8-FPDW model

**Figure 5:**
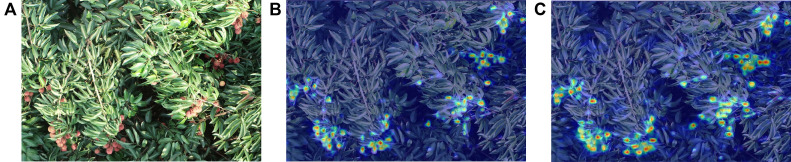
Comparison of visualization feature maps. **(A)** Test image. **(B)** Feature map of the YOLOv8 model. **(C)** Feature map of the proposed model.

The mAP@0.5 of YOLOv8-FPDW reached 87.7%, representing a 2.7% improvement over the original YOLOv8 (see **Table 1**; **Supplementary Figure S6** in the **Supplementary Material**). This performance enhancement was due to the combined effect of the FasterNet, ParNetAttention, DADet, and Wiou modules. FasterNet improved feature extraction efficiency, reducing the model size and complexity, while ParNetAttention enhanced feature expression capability. DADet optimized the detection head design, and Wiou improved localization accuracy through its dynamic focusing mechanism. The weight of the improved model was reduced to 5.2MB (a 17.5% decrease), while the parameter count and computational load were reduced by 19.0% and 9.9%, respectively, achieving both model lightweighting and efficiency. To validate the feature extraction capability of the improved model, we compared the visualized feature maps before and after the modification, as shown in **Figure 5**. It was found that the improved model extracted more prominent features compared to the original YOLOv8 model.

### 3.2 Comparative analysis of model performance across scenarios

#### 3.2.1 Sensitivity analysis of the model to fruit distribution density

**Figure 6:**
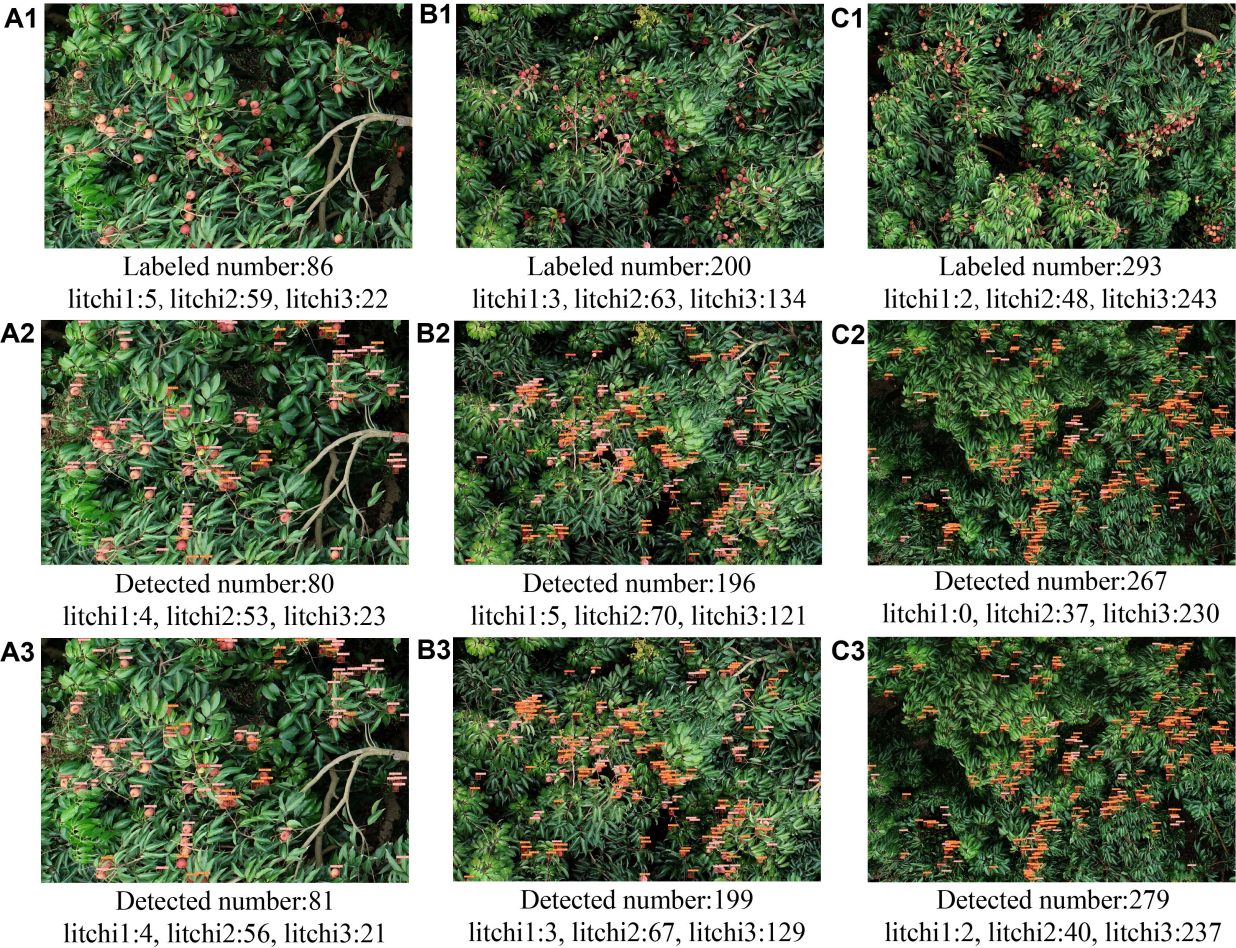
Detection results on litchi images with different distribution densities: **(A1, B1, C1)** original images showing low, medium, and high-density litchi distribution, respectively; **(A2, B2, C2)** detection results using the YOLOv8 algorithm; **(A3, B3, C3)** detection results using the YOLOv8-FPDW algorithm.

To evaluate the detection performance of the model before and after improvement under different target distribution densities, we randomly selected litchi canopy overhead images from low (**Figure 6A1**), medium (**Figure 6B1**), and high (**Figure 6C1**) density scenarios for testing, with target counts of 86, 200, and 293, respectively. The detection results were summarized in **Table 2**. In the low-density scenario, the error rate for litchi2 detection using YOLOv8 was 10.2% (**Figure 6 A2**), while YOLOv8-FPDW reduced it to 5.1% (**Figure 6 A3**), with errors for litchi1 and litchi3 remaining within one target. In the medium-density scenario, YOLOv8 showed a higher false detection rate, with litchi1, litchi2, and litchi3 having error rates of 20.0%, 10.0%, and 9.7%, respectively (**Figure 6 B2**). In contrast, YOLOv8-FPDW significantly reduced the false detection rates to 0%, 6.5%, and 3.7% (**Figure 6 B3**). In the high-density scenario, the performance of YOLOv8 significantly decreased, with no detections for litchi1 and detection rates for litchi2 and litchi3 showing high omission rates of 22.9% and 5.4%, respectively (**Figure 6 C2**). In contrast, YOLOv8-FPDW exhibited stronger robustness, reducing the error rates to 0%, 16.67%, and 1.65% (**Figure 6 C3**). Overall, YOLOv8-FPDW outperformed YOLOv8 in detection performance across different distribution densities, particularly in medium and high-density scenarios.

**Table 2:** Test results on litchi images with different distribution densities before and after model improvement.

Image	Litchi1			Litchi2			Litchi3		
	Detected	Real	Error rate	Detected	Real	Error rate	Detected	Real	Error rate
A2	4	5	20.0%	53	59	10.2%	23	22	4.5%
B2	5	3	66.7%	70	63	11.1%	121	134	9.7%
C2	0	2	100.0%	37	48	22.9%	230	243	5.3%
A3	4	5	20.0%	56	59	5.1%	21	22	4.5%
B3	3	3	0	67	63	6.3%	129	134	3.7%
C3	2	2	0	40	48	16.7%	237	243	2.5%

### 3.2.2 Performance comparison in diverse lighting and weather conditions

**Figure 7:**
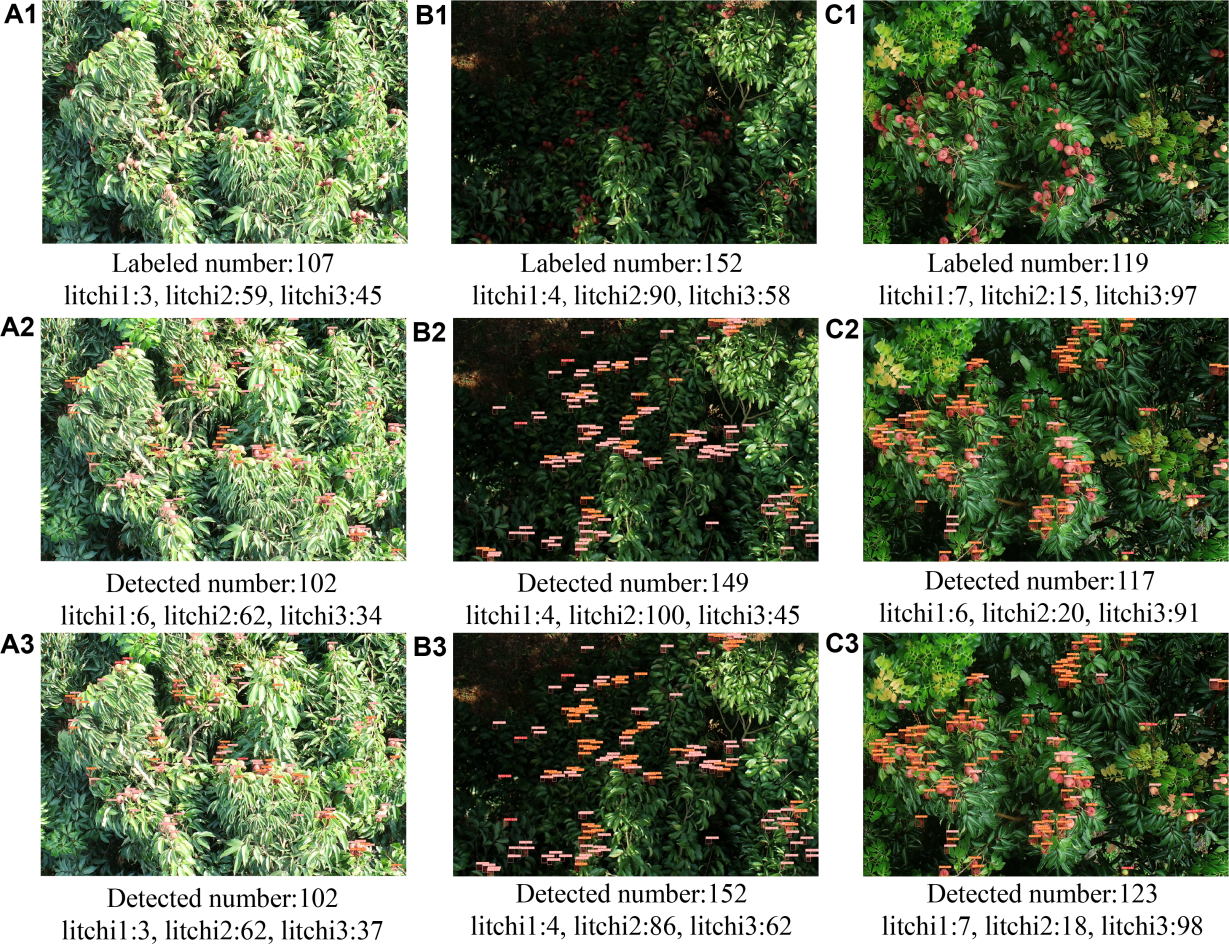
Detection results on litchi images in different scenes: **(A1, B1, C1)** original images are strong light, weak light, and rainy scenes, respectively; **(A2, B2, C2)** detection results using the YOLOv8algorithm; **(A3, B3, C3)** detection results using the YOLOv8-FPDWalgorithm.

To evaluate the adaptability of model in complex environments, three typical scenarios-strong light (**Figure 7A1**), low light (**Figure 7B1**), and rainy weather (**Figure 7C1**)-were selected to compare the detection performance of the original YOLOv8 and YOLOv8-FPDW models. The results were shown in **Table 3** and **Figure 7**. In the strong light scenario, YOLOv8 exhibited a high missed detection rate of 24.4% for litchi3, while YOLOv8-FPDW reduced this rate to 17.8%, and the error for litchi1 decreased from 3 to 0. In the low light scenario, YOLOv8 had error rates of 11.1% and 22.4% for litchi2 and litchi3, respectively, while YOLOv8-FPDW reduced these to 4.4% and 6.9%. In the rainy weather scenario, the error rates of YOLOv8 for all three types of targets were 14.3%, 33.3%, and 6.2%, while the improved model reduced these rates to 0%, 20%, and 1.0%, respectively. Notably, the improved model significantly reduced errors in detecting litchi1 and litchi3. Overall, YOLOv8-FPDW demonstrated higher robustness and detection accuracy in all three complex scenarios.

**Table 3:** Test results on litchi images in different scenarios before and after model improvement.

Image	Litchi1			Litchi2			Litchi3		
	Detected	Real	Error rate	Detected	Real	Error rate	Detected	Real	Error rate
A2	6	3	100.0%	62	59	5.1%	34	45	24.4%
B2	4	4	0	100	90	11.1%	45	58	22.4%
C2	6	7	12.3%	20	15	33.3%	91	97	6.2%
A3	3	3	0	62	59	5.1%	37	45	17.8%
B3	4	4	0	86	90	4.4%	62	58	6.9%
C3	7	7	0	18	15	20.0%	98	97	1.0%

### 3.3 Analysis of detection results for litchi at different maturity stages

**Figure 8:**
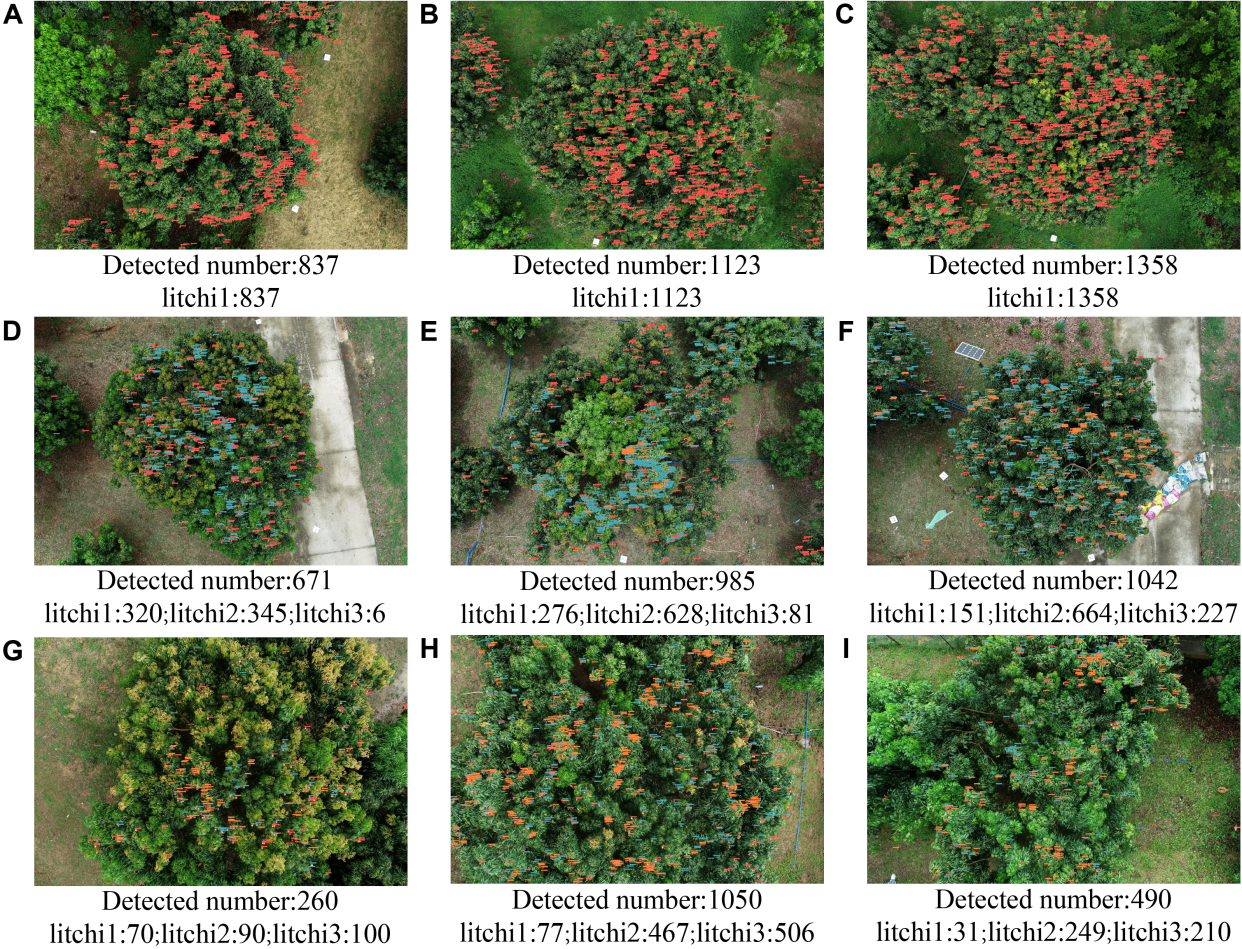
Detection results of the maturation state of litchi fruits at different growth stages: **(A–C)** are data from Node 2; **(D–F)** are data from Node 3; **(G–I)** are data from Node 4.

To investigate the distribution of litchi fruit maturity at different growth stages, the YOLOv8-FPDW model was used to perform inference on raw remote sensing images from Node 2 (June 1), Node 3 (June 14), and Node 4 (June 19). Due to the large size of the original images (5472×3648), the SAHI algorithm was employed to perform slice-based inference. The detection results, as shown in **Figure 8** and **Table 4**, revealed the distribution characteristics and dynamic changes of litchi fruits at various growth stages.

1. Early Rapid Development Stage (Node 2): As shown in **Figures 8A–C** and **Table 4**, in the three randomly selected images, all litchi fruits were immature (litchi1), with quantities of 837, 1123, and 1358 fruits, respectively.
2. Maturity State Differentiation Stage (Node 3): As shown in **Figures 8D–F** and **Table 4**, significant differentiation in the maturity state of the litchi fruits occurred during this stage. Litchi2 became the predominant category (51.42%, 63.75%, and 64.84%), litchi1 was the secondary category (47.69%, 28.02%, and 14.74%), and litchi3 had a low proportion (0.89%, 8.22%, and 22.17%). These data revealed a dynamic transformation trend of litchi fruit from immature (litchi1) to semi-mature (litchi2) states, and there were differences in the maturation process of the three trees.
3. Peak Maturity Stage (Node 4): As shown in **Figures 8G–I** and **Table 4**, the maturity of litchi fruits continued to progress, with the proportion of litchi1 significantly decreasing to 26.92%, 7.33%, and 6.33%. Litchi2 and litchi3 became the main components. On average, the proportions of litchi2 and litchi3 were similar, at 43.31% and 43.17%, respectively.

**Table 4:** Detection results of litchi fruit at different growth stages.

Image	Litchi1	Proportion	Litchi2	Proportion	Litchi3	Proportion	Overall
A	837	100%	–	–	–	–	837
B	1123	100%	–	–	–	–	1123
C	1385	100%	–	–	–	–	1358
D	320	47.69%	345	51.42%	6	0.89%	671
E	276	28.02%	628	63.75%	81	8.22%	985
F	151	14.74%	664	64.84%	227	22.17%	1024
G	70	26.92%	90	34.62%	100	38.46%	260
H	77	7.33%	467	44.48%	506	48.19%	1050
I	31	6.33%	249	50.82%	210	42.86%	490

### 3.4 Analysis and validation of target quantity differential strategy

#### 3.4.1 Impact of target quantity differential strategy on detection results

To address the issue of lower detection accuracy for litchi2 compared to litchi1 and litchi3 (80.5%, 91.3%, and 91.4%, respectively, see **Supplementary Table S1** in the **Supplementary Material**) in the improved YOLOv8-FPDW model, a target quantity differential strategy was proposed for calibration. By estimating the number of litchi2 fruits indirectly, based on the difference between the total number of targets detected by the YOLOv8-FPDW-all model and the number of litchi1 and litchi3 detected by the YOLOv8-FPDW model, calibration was performed. The calibrated detection results were shown in **Supplementary Table S2** in the **Supplementary Material**, which displayed the distribution and proportion of litchi targets in different maturity states at Node 3 (**Figures 8D–F**) and Node 4 (**Figures 8G–I**).

After calibration of the data for Node 3, both the number and proportion of litchi2 targets changed significantly. In **Figure 8D**, the number of litchi2 increased from 345 to 382, and its proportion rose to 53.95%. In **Figures 8E, F**, the proportions of litchi2 were adjusted from 63.75% and 64.84% to 66.19% and 63.05%, respectively. The calibration results indicated that litchi2 still dominated the total number of fruits at this stage.

For the data at Node 4, after calibration, the characteristics of litchi entering the peak maturity period became evident, with a significant increase in the proportion of litchi3. In **Figure 8H**, the number of litchi2 was calibrated to 418, and its proportion increased to 41.76%. The proportion of litchi3 rose to 50.55%, indicating that most of the fruits had matured. In **Figures 8G, I**, the proportion of litchi3 was calibrated to 39.68% and 44.21%, while the proportion of litchi1 increased to 27.78% and 6.53%, respectively. On average, the proportion of litchi2 was lower than that of litchi3, with values of 42.48% and 47.22%, respectively.

#### 3.4.2 Validation of target quantity differential strategy effectiveness

To verify the effectiveness of the differential strategy in improving the detection accuracy of litchi2 targets, we compared the performance of the YOLOv8-FPDW model with the YOLOv8-FPDW-all model based on the differential strategy in detecting the number of litchi2 targets. The experiment utilized 20 randomly selected test images, and the number of litchi2 targets detected by both methods was compared with the true values. The results were shown in **Supplementary Table S3** in the **Supplementary Material**.

The data in **Supplementary Table S3** indicated that the differential strategy improved the detection accuracy of litchi2 targets by adjusting the model output. For test images 7 and 12, for example, the error rates for YOLOv8-FPDW were 14.29% and 27.27%, respectively, whereas the differential strategy significantly reduced the error rates to 5.71% and 4.55%. By indirectly estimating the number of litchi2 targets, the differential strategy minimized errors caused by ambiguous classification boundaries, bringing the detection results closer to the true values. Overall, the average detection error for litchi2 targets across the 20 test images decreased by 12.58% compared to the YOLOv8-FPDW model.

### 3.5 Distribution characteristics of litchi maturity state at different growth stages

To quantify the distribution of litchi fruit maturity states at different growth stages, this study randomly selected 20 remote sensing images from Node 3 and Node 4, and analyzed the dynamic changes in maturation states based on the YOLOv8-FPDW model combined with the target differential strategy. The related results were shown in **Supplementary Tables S4** and **S5** in the **Supplementary Material**.

**Supplementary Table S4** showed that the average proportions of litchi1, litchi2, and litchi3 were 29.39%, 52.83%, and 17.78%, respectively, indicating that most of the litchi fruits were in the semi-mature stage, with a low proportion of mature fruits. It was noteworthy that litchi2 occupied more than 50% in most images, with the highest proportion reaching 70.18% (Test Image 8), reflecting the rapid transition of litchi from immature (litchi1) to semi-mature (litchi2) states.

**Supplementary Table S5** showed that the average proportion of litchi3 increased to 52.88%, a 35.1% increase from the previous node, indicating that most of the fruit had reached the mature stage. The proportions of litchi1 and litchi2 decreased to 7.31% and 39.82%, respectively. In individual images, the proportion of litchi3 exceeded 50% in most cases, with Test Images 10 and 12 reaching 63.35% and 59.83%, respectively. However, in Test Image 13, the proportion of litchi3 was 47.36%, indicating variability in the maturity progression across the trees.

Based on the data from these two nodes, it was evident that the maturation process of litchi accelerated significantly within 5 days. The average proportion of immature fruit (litchi1) decreased from 29.39% to 7.31%, while the proportion of mature fruit (litchi3) increased from 17.78% to 52.88%. Approximately 22% of litchi1 transitioned to litchi2, and 33% of litchi2 further transitioned to litchi3, indicating that litchi had entered the peak maturity stage.

### 3.6 Dynamic analysis of fruit ripening on individual trees at different growth stages

**Figure 9:**
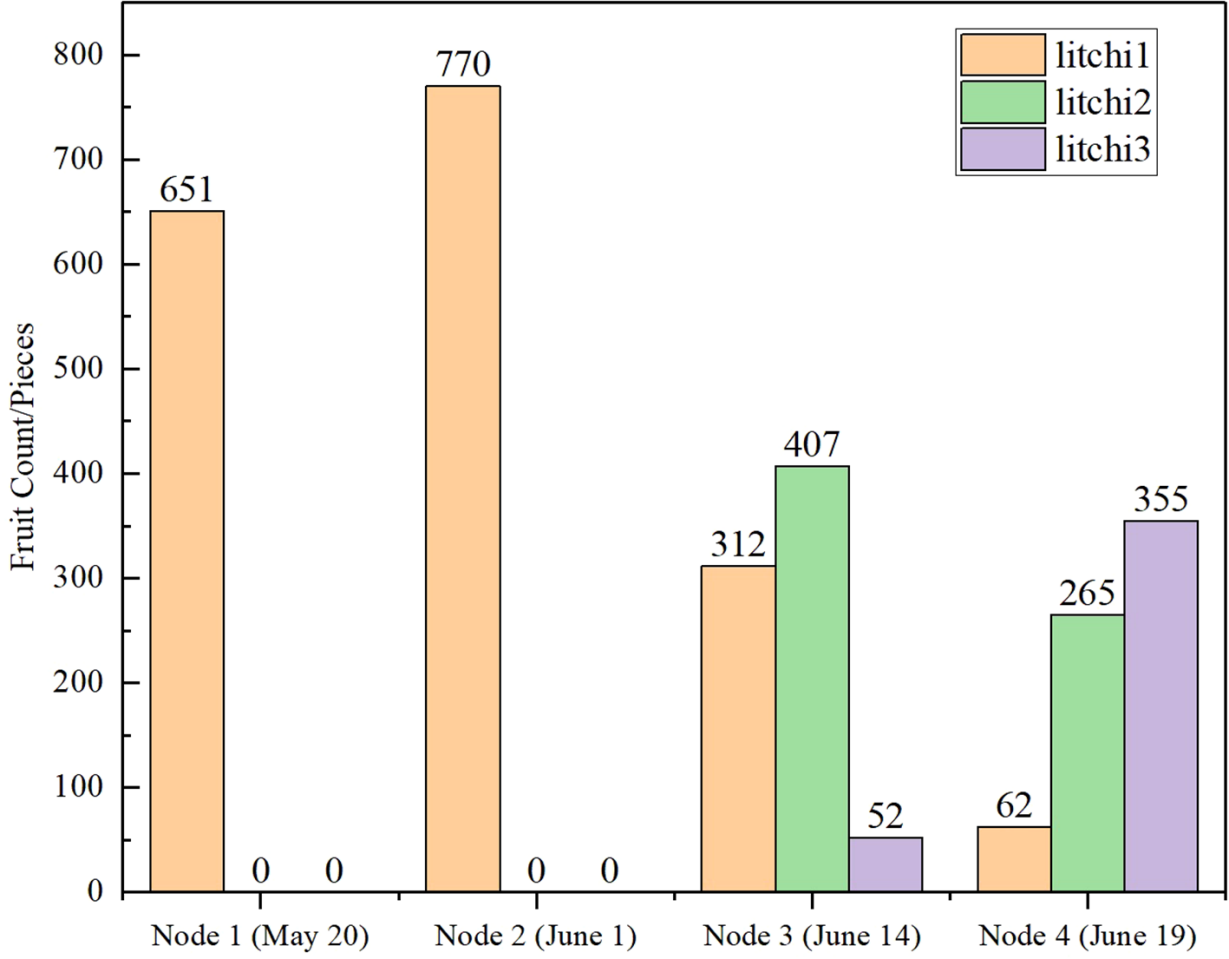
Average Fruit Count per Individual Tree at Different Growth Stages.

To verify whether the maturity dynamics of litchi fruits based on remote sensing images could be applied to the analysis of the maturity states on individual trees, this study randomly selected 20 trees and conducted long-term tracking analysis of fruit count and maturity state distribution using the YOLOv8-FPDW model combined with the target quantity differential strategy (relevant data are shown in **Figure 9** and **Supplementary Table S6** in the **Supplementary Material**).

The results showed that the ripening dynamics of individual trees were highly consistent with the patterns observed in remote sensing image analysis. At Node 1, the average fruit count per tree was 651, and all the fruits were immature (litchi1). By Node 2, the average fruit count increased to 770, an 18.28% growth, mainly due to the differentiation of the second batch of litchi flowers, which replenished the number of fruits. At this stage, the fruits were still primarily litchi1, with no obvious differentiation in ripening states. At Node 3, the fruit ripening states showed clear differentiation, and the average fruit count stabilized at 771, indicating that the differentiation of the second batch of litchi flowers had been basically completed. At this stage, the average proportions of litchi1, litchi2, and litchi3 were 40.45%, 52.82%, and 6.73%, respectively, with litchi2 becoming the dominant category, consistent with the proportion of litchi2 (52.83%) observed in remote sensing images. At Node 4, the tree entered the peak ripening period, and the average fruit count dropped to 682, a reduction of 11.46%, primarily due to physiological fruit drop (e.g., insufficient nutrition of small fruits) and physical drop caused by continuous rainfall. At this stage, the proportions of litchi1, litchi2, and litchi3 were 9.04%, 38.89%, and 52.06%, respectively, which closely matched the proportions in remote sensing images (7.31%, 39.82%, and 52.88%).

The long-term tracking study of individual trees further confirmed the regularity and applicability of the ripening dynamics observed in remote sensing image analysis. The ripening dynamics of litchi showed significant stage-based changes: during the rapid growth period (Node 1 to Node 2), the fruit count increased significantly; during the maturation differentiation period (Node 2 to Node 3), the proportion of semi-mature fruit (litchi2) increased rapidly and became dominant; during the peak ripening period (Node 3 to Node 4), the proportion of mature fruits (litchi3) surpassed 50%, marking the transition to the harvestable state for most litchi fruits. According to expert advice, the optimal harvest time was when the proportion of mature fruits (such as the varieties Xianjinfeng and Guiwei) approaches 65%. Based on the current ripening trend, it was predicted that the best harvest time for litchi would be around June 23, and the actual harvest period (from June 22 to June 26) further validated the accuracy of this prediction.

## 4 Discussion

### 4.1 Comparative analysis with mainstream detection models

The investigation above demonstrated that the YOLOv8-FPDW model achieved a 2.7% improvement in mAP@0.5, along with reductions in model weight (17.5%), parameter count (19.0%), and computational complexity (9.9%). These improvements resulted from the integration of modules such as FasterNet, ParNetAttention, DADet, and Wiou, which improved feature extraction, focused on key regions, optimized the detection head, and enhanced localization accuracy. The reductions in model weight and complexity enable more efficient deployment of the model in resource-constrained agricultural environments, such as mobile devices and low-cost platforms. The detection error rates of YOLOv8-FPDW were reduced by 55.56%, 5.36%, and 2.93% for litchi1, litchi2, and litchi3, respectively, in varying densities, and 37.63%, 6.67%, and 9.10% in complex conditions, demonstrating its potential for agricultural applications. Additionally, the proposed target quantity differential strategy significantly improved the detection accuracy of litchi2, reducing the error rate by 12.58%, providing valuable insights for similar maturity classification tasks in agricultural scenarios where targets are in transitional stages and difficult to classify accurately.

Further analysis was conducted to compare the performance of the YOLOv8-FPDW algorithm with five mainstream detection algorithms: YOLOv5s, YOLOv7 (Wang et al., 2023), YOLOv8n, YOLOv10n (Wang et al., 2024), and YOLOv11n (Khanam and Hussain, 2024) (as detailed in **Table 5**). To ensure a fair comparison, the same training configuration (e.g., number of training epochs, batch size) was used, with other hyperparameters set to the default values of each algorithm.

**Table 5:** Performance comparison of YOLOv8-FPDW with mainstream detection algorithms.

Method	mAP@0.5(%)	mAP@0.95(%)	Weight	GFLOPs
YOLOv5s	84.5	58.6	14.8Mb	15.8
YOLOv7	86.5	59.5	75.0Mb	105.1
YOLOv8n	85.0	65.4	6.3Mb	8.1
YOLOv10n	85.2	60.1	5.9Mb	8.2
YOLOv11n	84.9	61.2	5.6Mb	**6.5**
YOLOv8-FPDW	**87.7**	**67.6**	**5.2Mb**	7.3

Bold values indicate the optimal results in each column.

Compared to the mainstream algorithms, YOLOv8-FPDW demonstrated clear advantages. In terms of recognition accuracy, YOLOv8-FPDW improved by 3.2%, 2.5%, and 2.8% over YOLOv5s (84.5%), YOLOv10n (85.2%), and YOLOv11n (84.9%), respectively. In terms of model lightweighting, YOLOv8-FPDW had the smallest model weight at 5.2 MB. Regarding computational complexity (GFLOPs), YOLOv8-FPDW required only 7.3 GFLOPs, which represented a reduction of 11.0% and 12.3% compared to YOLOv8n (8.1 GFLOPs) and YOLOv10n (8.2 GFLOPs), respectively, indicating lower computational resource demands. Although it required slightly more GFLOPs than YOLOv11n (6.5 GFLOPs), the recognition accuracy of YOLOv8-FPDW was significantly higher than that of YOLOv11n (84.9%). In conclusion, the YOLOv8-FPDW model achieved a good balance between accuracy, model lightweighting, and computational complexity, making it suitable for deployment on mobile devices.

### 4.2 Limitations and challenges in practical application

Although the YOLOv8-FPDW model was more competitive than mainstream detection algorithms in detecting litchi fruits at different maturity stages, some limitations occurred for the proposed approach in practical application. First, the detection accuracy might decrease when images were collected at a much strong or weak sunlight condition. This issue could be mitigated through additional preprocessing techniques, such as illumination correction and image enhancement. Second, due to the perspective limitations of drone imaging, parts of fruits on the sides of tree canopy could not be captured in drone-based remote sensing images. By integrating data from both drones and ground sensors, it would have been possible to detect fruits on both the top and sides of the canopy, and providing a method for counting the total number of fruits on the outer canopy. Additionally, parts of litchi fruits located within the inner canopy were completely hidden by foliage and could not be detected by the UAV-based approach. This limitation was a common challenge of visual detection techniques and could have caused an underestimation of the total fruit count per tree and lower the yield estimation accuracy. Ground verification methods, such as manual counting, could be used to calibrate the model outputs and establish the correlation between visible fruits and total yield.

### 4.3 Future research directions

In the future, we will further optimize the litchi maturity detection method to improve the applicability and accuracy of the model. At the image level, we aim to develop higher-precision and more robust artificial intelligence models to adapt to fruit detection tasks in natural environments. Additionally, we intend to investigate the adaptability of the model at higher flight altitudes, combining super-resolution reconstruction techniques to enhance its detection capability in low-resolution images. At tree level, new approaches will be developed to estimate the total fruit count of a tree, such as the systematic sampling method proposed by Wulfsohn et al. (2012), which includes both the fruits at outer and inner tree canopy. Furthermore, we will establish machine learning regression models linking the total number of fruits and the number identified by the drone method, providing more precise individual tree-level yield estimates for orchard management. At the orchard level, we will integrate the drone-based fruit counting method with the established machine learning regression models to develop high-precision orchard yield estimation methods. By incorporating the dynamic distribution characteristics of fruit maturity within the region, orchard management strategies can be optimized.

## 5 Conclusion

This study proposed a litchi fruit maturity state detection method based on UAV remote sensing technology and YOLOv8-FPDW. The YOLOv8-FPDW model demonstrated significant advantages, with a 2.7% improvement in detection accuracy, reaching 87.7%, and achieved reductions in model weight, parameter count, and computational complexity by 17.5%, 19.0%, and 9.9%, respectively. The improved model showed strong robustness across various scenarios. The introduced differential strategy significantly enhanced the detection accuracy for litchi2, reducing the error by 12.58%. Analysis of remote sensing images revealed the stage-wise dynamic changes in the maturity state of litchi fruits. From Node 3 to Node 4, the proportion of litchi1 dropped significantly from 29.39% to 7.31%, the proportion of litchi2 decreased from 52.83% to 39.81%, while the proportion of mature fruits (litchi3) rapidly increased to 52.88%. Long-term tracking of individual trees further confirmed this trend. During the rapid growth phase, the fruit count increased by 18.28%. In the maturity differentiation phase, litchi2 became the dominant category, accounting for approximately 53% of the total. During the maturity peak phase, the proportion of litchi3 exceeded 50%, and the fruit drop rate reached 11.46%. Additionally, compared with mainstream object detection algorithms, YOLOv8-FPDW demonstrated superior detection accuracy and model weight efficiency. Future work will focus on enhancing the adaptability of the YOLOv8-FPDW model to diverse orchard environments and integrating advanced methods, such as super-resolution reconstruction, to improve detection accuracy for low-resolution targets, supporting more precise orchard management.

## Data Availability

The raw data supporting the conclusions of this article will be made available by the authors, without undue reservation.

## References

[B1] Akyon F. C. Altinuc S. O. Temizel A. (2022). “Slicing aided hyper inference and fine-tuning for small object detection,” in 2022 IEEE International Conference on Image Processing (ICIP), IEEE, 966–970. doi: 10.1109/ICIP46576.2022.9897990

[B2] Apolo-Apolo O. E. Martínez-Guanter J. Egea G. Raja P. Pérez-Ruiz M. (2020). Deep learning techniques for estimation of the yield and size of citrus fruits using a UAV. Eur. J. Agron. 115, 126030. doi: 10.1016/j.eja.2020.126030

[B3] Bouguettaya A. Zarzour H. Taberkit A. M. Kechida A. (2022). A review on early wildfire detection from unmanned aerial vehicles using deep learning-based computer vision algorithms. Signal Process. 190, 108309. doi: 10.1016/j.sigpro.2021.108309

[B4] Chen J. Kao S. H. He H. Zhuo W. Wen S. Lee C. H.. (2023). “Run, don’t walk: chasing higher FLOPS for faster neural networks,” in Proceedings of the IEEE/CVF conference on computer vision and pattern recognition, IEEE, 12021–12031. doi: 10.1109/CVPR52729.2023.01157

[B5] Chen W. Liu M. Zhao C. Li X. Wang Y. (2024). MTD-YOLO: Multi-task deep convolutional neural network for cherry tomato fruit bunch maturity detection. Comput. Electron. Agric. 216, 108533. doi: 10.1016/j.compag.2023.108533

[B6] Gao F. Fu L. Zhang X. Majeed Y. Li R. Karkee M.. (2020). Multi-class fruit-on-plant detection for apple in SNAP system using Faster R-CNN. Comput. Electron. Agric. 176, 105634. doi: 10.1016/j.compag.2020.105634

[B7] Gené-Mola J. Sanz-Cortiella R. Rosell-Polo J. R. Morros J. R. Ruiz-Hidalgo J. Vilaplana V.. (2020). Fruit detection and 3D location using instance segmentation neural networks and structure-from-motion photogrammetry. Comput. Electron. Agric. 169, 105165. doi: 10.1016/j.compag.2019.105165

[B8] Ghasemi Y. Jeong H. Choi S. H. Park K. B. Lee J. Y. (2022). Deep learning-based object detection in augmented reality: A systematic review. Comput. Industry 139, 103661. doi: 10.1016/j.compind.2022.103661

[B9] Girshick R. (2015). “Fast r-cnn,” in Proceedings of the IEEE international conference on computer vision, IEEE, 1440–1448. doi: 10.1109/ICCV.2015.169

[B10] Girshick R. Donahue J. Darrell T. Malik J. (2014). “Rich feature hierarchies for accurate object detection and semantic segmentation,” in Proceedings of the IEEE conference on computer vision and pattern recognition, IEEE, 580–587. doi: 10.1109/CVPR.2014.81

[B11] Goyal A. Bochkovskiy A. Deng J. Koltun V. (2022). Non-deep networks. Adv. Neural Inf. Process. Syst. 35, 6789–6801.

[B12] Hadas E. Jozkow G. Walicka A. Borkowski A. (2019). Apple orchard inventory with a LiDAR equipped unmanned aerial system. Int. J. Appl. Earth Observation Geoinformation 82, 101911. doi: 10.1016/j.jag.2019.101911

[B13] Khanam R. Hussain M. (2024). Yolov11: An overview of the key architectural enhancements. arXiv. doi: 10.48550/arXiv.2410.17725

[B14] Li C. Lin J. Li B. Zhang S. Li J. (2022). Partition harvesting of a column-comb litchi harvester based on 3D clustering. Comput. Electron. Agric. 197, 106975. doi: 10.1016/j.compag.2022.106975

[B15] Li C. Lin J. Li Z. Mai C. Jiang R. Li J. (2024). An efficient detection method for litchi fruits in a natural environment based on improved YOLOv7-Litchi. Comput. Electron. Agric. 217, 108605. doi: 10.1016/j.compag.2023.108605

[B16] Li C. Zhang Y. Qu Y. (2018). “Object detection based on deep learning of small samples,” in 2018 IEEE Tenth International Conference on Advanced Computational Intelligence (ICACI), IEEE, 449–454. doi: 10.1109/ICACI.2018.8377501

[B17] Liang J. Chen X. Liang C. Long T. Tang X. Shi Z.. (2023). A detection approach for late-autumn shoots of litchi based on unmanned aerial vehicle (UAV) remote sensing. Comput. Electron. Agric. 204, 107535. doi: 10.1016/j.compag.2022.107535

[B18] Liang C. Liang J. Yang W. Ge W. Zhao J. Li Z.. (2025). Enhanced visual detection of litchi fruit in complex natural environments based on unmanned aerial vehicle (UAV) remote sensing. Precis. Agric. 26, 23. doi: 10.1007/s11119-025-10220-w

[B19] Lin T. Y. Goyal P. Girshick R. He K. Dollár P. (2017). “Focal loss for dense object detection,” in IEEE Transactions on Pattern Analysis and Machine Intelligence, IEEE. 42, 318–327. doi: 10.1109/TPAMI.2018.2858826 30040631

[B20] Lin P. Li D. Jia Y. Chen Y. Huang G. Elkhouchlaa H.. (2022). A novel approach for estimating the flowering rate of litchi based on deep learning and UAV images. Front. Plant Sci. 3001. doi: 10.3389/fpls.2022.966639 PMC9453484 36092399

[B21] Liu W. Anguelov D. Erhan D. Szegedy C. Reed S. Fu C. Y.. (2016). “Ssd: Single shot multibox detector,” in Proceedings of the European conference on computer vision (ECCV), ECCV, 21–37. doi: 10.1007/978-3-319-46448-0_2

[B22] Liu G. Nouaze J. C. Touko Mbouembe P. L. Kim J. H. (2020). YOLO-tomato: A robust algorithm for tomato detection based on YOLOv3. Sensors 20, 2145. doi: 10.3390/s20072145 32290173 PMC7180616

[B23] Liu Y. Zheng H. Zhang Y. Zhang Q. Chen H. Xu X.. (2023). Is this blueberry ripe?”: a blueberry ripeness detection algorithm for use on picking robots. Front. Plant Sci. 14. doi: 10.3389/fpls.2023.1198650 PMC10289036 37360727

[B24] Maity M. Banerjee S. Chaudhuri S. S. (2021). “Faster r-cnn and yolo based vehicle detection: A survey,” in 2021 IEEE International Conference on Computing Methodologies and Communication (ICCMC), IEEE, 1442–1447. doi: 10.1109/ICCMC51019.2021.9418274

[B25] Modica G. Messina G. De Luca G. Fiozzo V. Praticò S. (2020). Monitoring the vegetation vigor in heterogeneous citrus and olive orchards. A multiscale object-based approach to extract trees’ crowns from UAV multispectral imagery. Comput. Electron. Agric. 175, 105500. doi: 10.1016/j.compag.2020.105500

[B26] Nan Y. Zhang H. Zeng Y. Zheng J. Ge Y. (2023). Intelligent detection of Multi-Class pitaya fruits in target picking row based on WGB-YOLO network. Comput. Electron. Agric. 208, 107780. doi: 10.1016/j.compag.2023.107780

[B27] Redmon J. Divvala S. Girshick R. Farhadi A. (2016). “You only look once: Unified, real-time object detection,” in 2016 IEEE Conference on Computer Vision and Pattern Recognition (CVPR), IEEE, 779–788. doi: 10.1109/CVPR.2016.91

[B28] Ren S. He K. Girshick R. Sun J. (2017). Faster R-CNN: Towards real-time object detection with region proposal networks. IEEE Trans. Pattern Anal. Mach. Intell. 39, 1137–1149. doi: 10.1109/TPAMI.2016.2577031 27295650

[B29] Tian Y. Yang G. Wang Z. Wang H. Li E. Liang Z. (2019). Apple detection during different growth stages in orchards using the improved YOLO-V3 model. Comput. Electron. Agric. 157, 417–426. doi: 10.1016/j.compag.2019.01.012

[B30] Tong Z. Chen Y. Xu Z. Yu R. (2023). Wise-IoU: bounding box regression loss with dynamic focusing mechanism. arXiv. doi: 10.48550/arXiv.2301.10051

[B31] Vasconez J. P. Delpiano J. Vougioukas S. Cheein F. A. (2020). Comparison of convolutional neural networks in fruit detection and counting: A comprehensive evaluation. Comput. Electron. Agric. 173, 105348. doi: 10.1016/j.compag.2020.105348

[B32] Wang C. Y. Bochkovskiy A. Liao H. Y. M. (2023). “YOLOv7: Trainable bag-of-freebies sets new state-of-the-art for real-time object detectors,” in Proceedings of the IEEE/CVF conference on computer vision and pattern recognition, IEEE, 7464–7475.

[B33] Wang A. Chen H. Liu L. Chen K. Lin Z. Han J.. (2024). Yolov10: Real-time end-to-end object detection. Adv. Neural Inf. Process. Syst. 37, 107984–108011. doi: 10.48550/arXiv.2405.14458

[B34] Wang Y. Yan G. Meng Q. Yao T. Han J. Zhang B. (2022a). DSE-YOLO: Detail semantics enhancement YOLO for multi-stage strawberry detection. Comput. Electron. Agric. 198, 107057. doi: 10.1016/j.compag.2022.107057

[B35] Wang L. Zhao Y. Xiong Z. Wang S. Li Y. Lan Y. (2022b). Fast and precise detection of litchi fruits for yield estimation based on the improved YOLOv5 model. Front. Plant Sci. 13. doi: 10.3389/fpls.2022.965425 PMC9396223 36017261

[B36] Wei Y. Zhang H. Li W. Xie J. Wang Y. Liu L.. (2013). Phenological growth stages of lychee (Litchi chinensis Sonn.) using the extended BBCH-scale. Scientia Hortic. 161, 273–277. doi: 10.1016/j.scienta.2013.07.017

[B37] Wu J. Yang G. Yang H. Zhu Y. Li Z. Lei L.. (2020). Extracting apple tree crown information from remote imagery using deep learning. Comput. Electron. Agric. 174, 105504. doi: 10.1016/j.compag.2020.105504

[B38] Wu J. Zhang S. Zou T. Dong L. Peng Z. Wang H. (2022). A dense litchi target recognition algorithm for large scenes. Math. Problems Eng. 2022, 4648105. doi: 10.1155/2022/4648105

[B39] Wulfsohn D. Aravena Zamora F. Potin Téllez C. Zamora Lagos I. García-Fiñana M. (2012). Multilevel systematic sampling to estimate total fruit number for yield forecasts. Precis. Agric. 13, 256–275. doi: 10.1007/s11119-011-9245-2

[B40] Xie J. Peng J. Wang J. Chen B. Jing T. Sun D.. (2022). Litchi detection in a complex natural environment using the YOLOv5-litchi model. Agronomy 12, 3054. doi: 10.3390/agronomy12123054

[B41] Xiong Z. Wang L. Zhao Y. Lan Y. (2023). Precision detection of dense litchi fruit in UAV images based on improved YOLOv5 model. Remote Sens. 15, 4017. doi: 10.3390/rs15164017

[B42] Yang S. Wang W. Gao S. Deng Z. (2023). Strawberry ripeness detection based on YOLOv8 algorithm fused with LW-Swin Transformer. Comput. Electron. Agric. 215, 108360. doi: 10.1016/j.compag.2023.108360

[B43] Zhang C. Serra S. Quirós-Vargas J. Sangjan W. Musacchi S. Sankaran S. (2023). Non-invasive sensing techniques to phenotype multiple apple tree architectures. Inf. Process. Agric. 10, 136–147. doi: 10.1016/j.inpa.2021.02.001

[B44] Zhang C. Valente J. Kooistra L. Guo L. Wang W. (2021). Orchard management with small unmanned aerial vehicles: A survey of sensing and analysis approaches. Precis. Agric. 22, 2007–2052. doi: 10.1007/s11119-021-09813-y

[B45] Zhao Z. Q. Zheng P. Xu S. T. Wu X. (2019). Object detection with deep learning: A review. IEEE Trans. Neural Networks Learn. Syst. 30, 3212–3232. doi: 10.1109/TNNLS.2018.2876865 30703038

