# Supplementary material for: Detection of litchi fruit maturity states based on unmanned aerial vehicle remote sensing and improved YOLOv8 model
Source: Front Plant Sci. 2025 Apr 16;16:1568237. doi: 10.3389/fpls.2025.1568237 (PMC12042761; doi:10.3389/fpls.2025.1568237)
Supplement: Supplementary file 1 [file DataSheet1.docx]

**Supplementary Material**


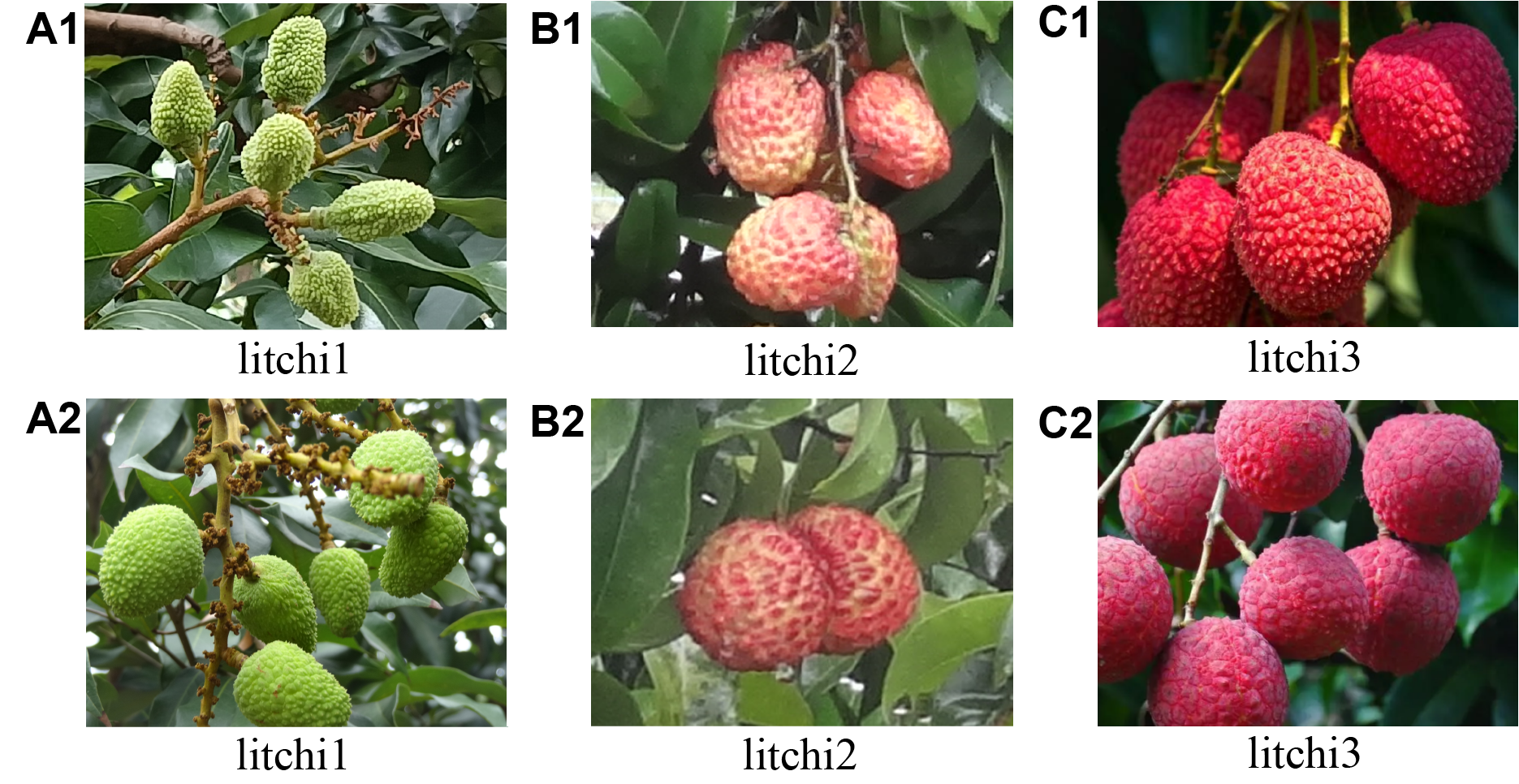


**Figure S1.** Illustration of litchi fruits at different maturity states


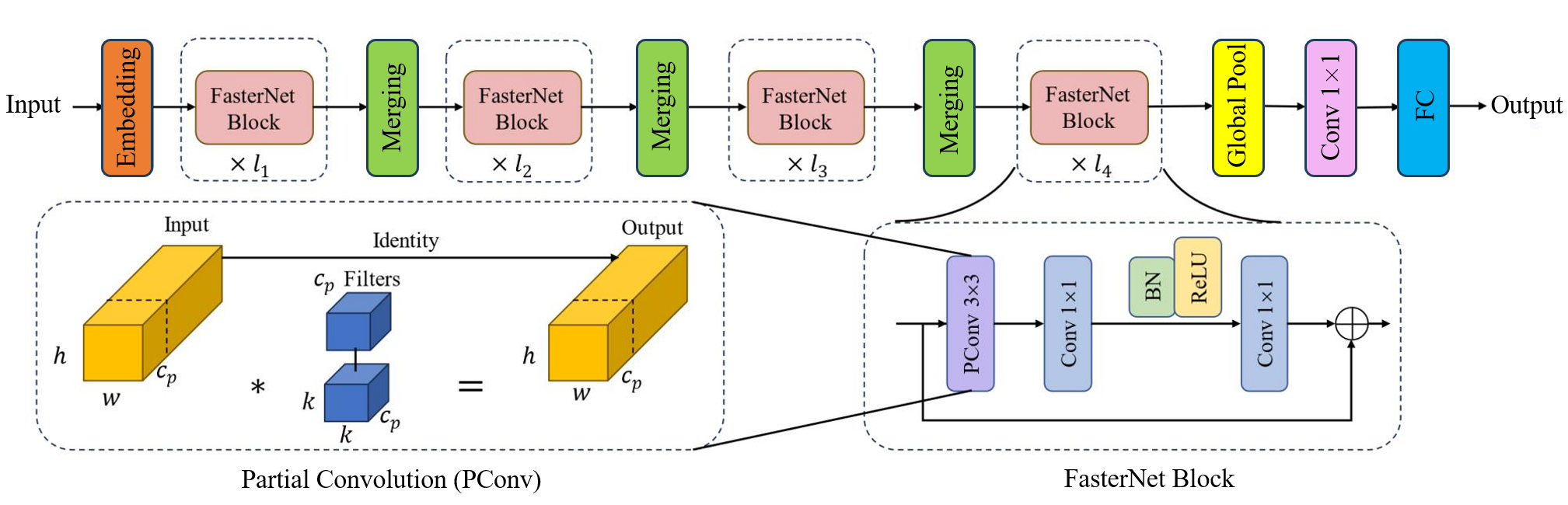


**Figure S2.** The network structure of FasterNet


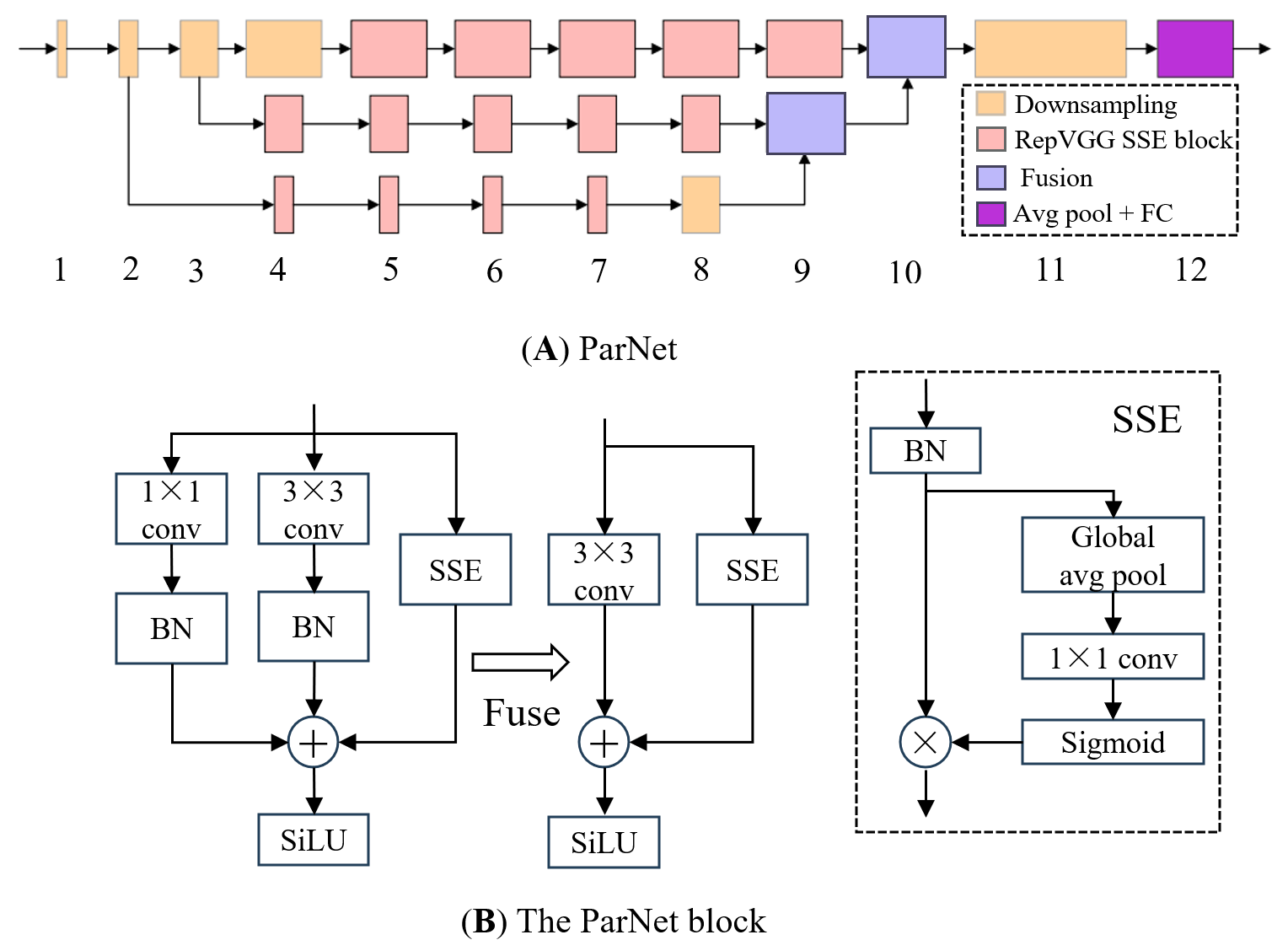


**Figure S3.** The network structure of ParNet (**A**) and ParNetAttention (**B**)


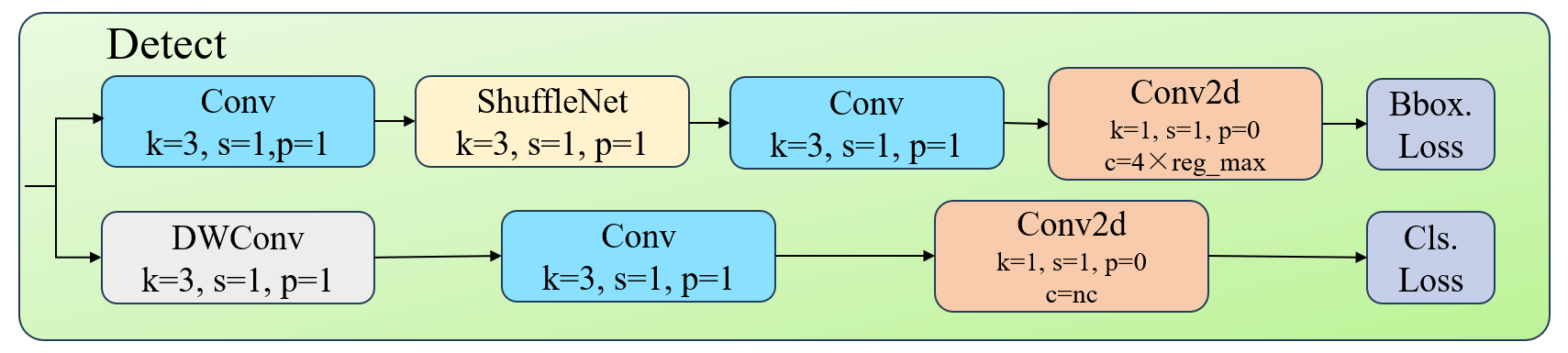


**Figure S4.** The network structure of DADet


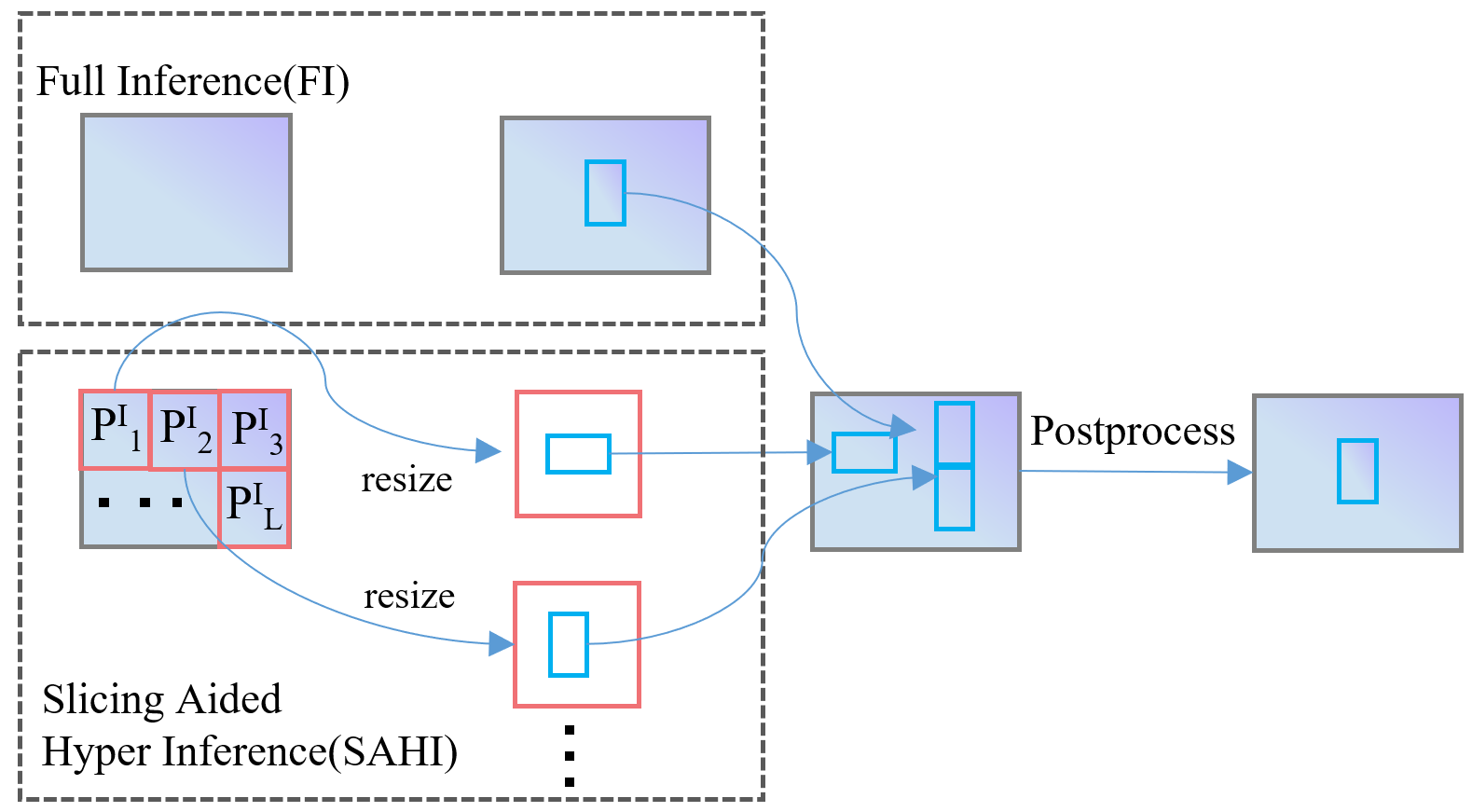


**Figure S5.** Slicing Aided Hyper Inference schematic


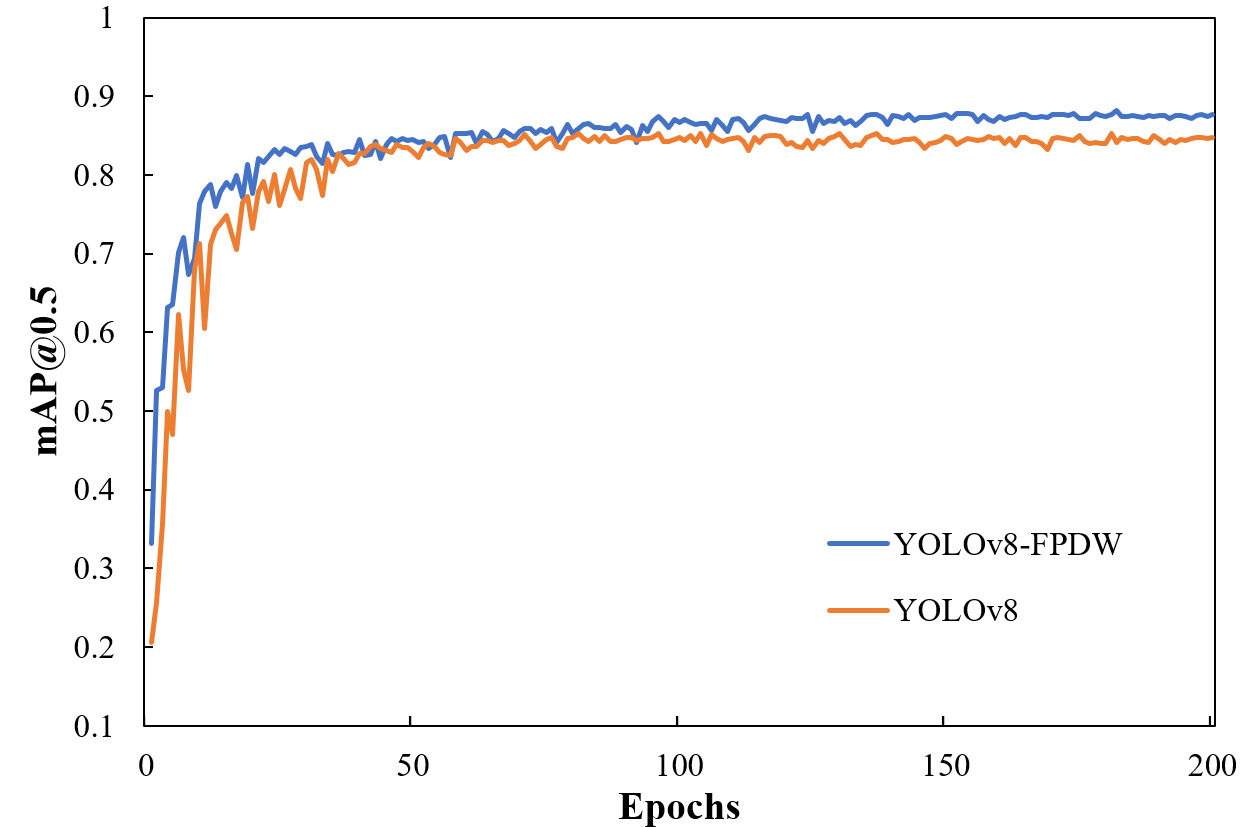


**Figure S6.** The mAP@0.5 curves of the model during the training process

**Table S1.** Evaluation of detection results of litchi at different maturity states before and after model improvement

| Model | all | litchi1 | litchi2 | litchi3 | Weight |
| --- | --- | --- | --- | --- | --- |
| YOLOv8 | 85.0% | 88.4% | 78.1% | 88.6% | 6.3Mb |
| YOLOv8-FPDW | 87.7% | 91.3% | 80.5% | 91.4% | 5.2Mb |

**Table S2.** Statistics of fruits at different maturity states based on target quantity difference strategy

| Image | litchi1 | Proportion | litchi2 | Proportion | litchi3 | Proportion | Overall |
| --- | --- | --- | --- | --- | --- | --- | --- |
| D | 320 | 45.20% | 382 | 53.95% | 6 | 0.85% | 708 |
| E | 276 | 26.14% | 699 | 66.19% | 81 | 8.22% | 1056 |
| F | 151 | 14.76% | 645 | 63.05% | 227 | 22.19% | 1023 |
| G | 70 | 27.78% | 82 | 32.54% | 100 | 39.68% | 252 |
| H | 77 | 7.69% | 418 | 41.76% | 506 | 50.55% | 1001 |
| I | 31 | 6.53% | 234 | 49.26% | 210 | 44.21% | 475 |

**Table S3.** Comparison of litchi2 target detection results using YOLOv8-FPDW and differential strategy

| Image | v8-FPDW | Differential Strategy | Real | Image | v8-FPDW | Differential Strategy | Real |
| --- | --- | --- | --- | --- | --- | --- | --- |
| 1 | 36 | 31 | 30 | 11 | 43 | 42 | 40 |
| 2 | 48 | 47 | 48 | 12 | 28 | 23 | 22 |
| 3 | 44 | 39 | 41 | 13 | 22 | 17 | 19 |
| 4 | 17 | 12 | 11 | 14 | 21 | 20 | 22 |
| 5 | 7 | 7 | 6 | 15 | 34 | 34 | 31 |
| 6 | 13 | 11 | 12 | 16 | 20 | 17 | 18 |
| 7 | 40 | 37 | 35 | 17 | 32 | 21 | 20 |
| 8 | 19 | 17 | 15 | 18 | 19 | 11 | 13 |
| 9 | 21 | 20 | 18 | 19 | 33 | 36 | 35 |
| 10 | 8 | 8 | 8 | 20 | 36 | 31 | 33 |

**Table S4.** Statistical results of different maturity states of litchi identification data (data from June 14)

| Image | litchi1 | Proportion | litchi2 | Proportion | litchi3 | Proportion | Overall |
| --- | --- | --- | --- | --- | --- | --- | --- |
| 1 | 188 | 31.70% | 253 | 42.66% | 152 | 25.63% | 593 |
| 2 | 162 | 28.72% | 315 | 55.85% | 87 | 15.43% | 564 |
| 3 | 183 | 31.88% | 266 | 46.34% | 125 | 21.78% | 574 |
| 4 | 268 | 32.76% | 402 | 49.14% | 148 | 18.09% | 818 |
| 5 | 308 | 31.88% | 490 | 50.72% | 168 | 17.39% | 966 |
| 6 | 204 | 30.13% | 372 | 54.95% | 101 | 14.92% | 677 |
| 7 | 289 | 38.53% | 365 | 48.67% | 96 | 12.80% | 750 |
| 8 | 210 | 20.67% | 713 | 70.18% | 93 | 9.15% | 1016 |
| 9 | 243 | 26.85% | 524 | 57.90% | 138 | 15.25% | 905 |
| 10 | 202 | 26.23% | 378 | 49.09% | 190 | 24.68% | 770 |
| 11 | 150 | 24.83% | 291 | 48.18% | 163 | 26.99% | 604 |
| 12 | 216 | 38.10% | 274 | 48.32% | 77 | 13.58% | 567 |
| 13 | 277 | 30.51% | 565 | 62.22% | 66 | 7.27% | 908 |
| 14 | 174 | 26.52% | 350 | 53.35% | 132 | 20.12% | 656 |
| 15 | 262 | 32.07% | 396 | 48.47% | 159 | 19.46% | 817 |
| 16 | 274 | 27.10% | 513 | 50.74% | 224 | 22.16% | 1011 |
| 17 | 255 | 27.66% | 525 | 56.94% | 142 | 15.40% | 922 |
| 18 | 433 | 35.17% | 697 | 56.62% | 101 | 8.20% | 1231 |
| 19 | 255 | 24.64% | 545 | 52.66% | 235 | 22.71% | 1035 |
| 20 | 190 | 21.91% | 464 | 53.52% | 213 | 24.57% | 867 |

**Table S5.** Statistical results of different maturity states of litchi identification data (data from June 19)

| Image | litchi1 | Proportion | litchi2 | Proportion | litchi3 | Proportion | Overall |
| --- | --- | --- | --- | --- | --- | --- | --- |
| 1 | 77 | 14.18% | 169 | 31.12% | 297 | 54.70% | 543 |
| 2 | 51 | 11.38% | 183 | 40.85% | 214 | 47.77% | 448 |
| 3 | 48 | 7.57% | 295 | 46.53% | 291 | 45.90% | 634 |
| 4 | 33 | 4.95% | 245 | 36.79% | 388 | 58.26% | 666 |
| 5 | 35 | 3.76% | 396 | 42.53% | 500 | 53.71% | 931 |
| 6 | 77 | 7.33% | 467 | 44.48% | 506 | 48.19% | 1050 |
| 7 | 65 | 8.35% | 372 | 47.81% | 341 | 43.83% | 778 |
| 8 | 72 | 12.50% | 260 | 45.14% | 244 | 42.36% | 576 |
| 9 | 52 | 10.86% | 171 | 35.70% | 256 | 53.44% | 479 |
| 10 | 31 | 4.29% | 234 | 32.37% | 458 | 63.35% | 723 |
| 11 | 55 | 5.68% | 345 | 35.64% | 568 | 58.68% | 968 |
| 12 | 35 | 3.23% | 400 | 36.93% | 648 | 59.83% | 1083 |
| 13 | 57 | 8.60% | 292 | 44.04% | 314 | 47.36% | 663 |
| 14 | 45 | 6.47% | 298 | 42.88% | 352 | 50.65% | 695 |
| 15 | 51 | 6.85% | 333 | 44.70% | 361 | 48.46% | 745 |
| 16 | 25 | 4.59% | 181 | 33.21% | 339 | 62.20% | 545 |
| 17 | 76 | 10.57% | 328 | 45.62% | 315 | 43.81% | 719 |
| 18 | 60 | 4.91% | 502 | 41.05% | 661 | 54.05% | 1223 |
| 19 | 42 | 4.61% | 319 | 34.98% | 551 | 60.42% | 912 |
| 20 | 56 | 5.50% | 346 | 33.95% | 617 | 60.55% | 1019 |

**Table S6.** Results of canopy fruit identification of individual fruit trees at different growth stages

| Fruit trees | Node 1 (May 20) | Node 2 (June 1) | Node 3 (June 14) | | | | Node 4 (June 19) | | | |
| --- | --- | --- | --- | --- | --- | --- | --- | --- | --- | --- |
|  | litchi1 | litchi1 | litchi1 | litchi2 | litchi3 | Overall | litchi1 | litchi2 | litchi3 | Overall |
| 1 | 224 | 248 | 215 | 80 | 34 | 329 | 70 | 91 | 101 | 262 |
| 2 | 460 | 533 | 145 | 302 | 59 | 506 | 31 | 189 | 256 | 476 |
| 3 | 636 | 660 | 176 | 351 | 14 | 541 | 13 | 187 | 247 | 447 |
| 4 | 545 | 646 | 309 | 369 | 29 | 707 | 48 | 372 | 317 | 737 |
| 5 | 563 | 648 | 286 | 318 | 81 | 685 | 79 | 301 | 284 | 664 |
| 6 | 256 | 284 | 147 | 134 | 27 | 308 | 61 | 120 | 146 | 327 |
| 7 | 624 | 775 | 267 | 338 | 58 | 663 | 76 | 324 | 252 | 652 |
| 8 | 906 | 1048 | 528 | 526 | 39 | 1093 | 91 | 330 | 427 | 848 |
| 9 | 526 | 588 | 494 | 166 | 4 | 664 | 125 | 259 | 199 | 583 |
| 10 | 534 | 646 | 175 | 490 | 86 | 751 | 70 | 262 | 332 | 664 |
| 11 | 649 | 963 | 268 | 578 | 105 | 951 | 51 | 275 | 595 | 921 |
| 12 | 948 | 1304 | 564 | 687 | 28 | 1279 | 112 | 404 | 526 | 1042 |
| 13 | 618 | 778 | 532 | 242 | 3 | 777 | 59 | 352 | 353 | 764 |
| 14 | 926 | 1078 | 159 | 720 | 100 | 979 | 61 | 308 | 501 | 870 |
| 15 | 1029 | 1139 | 342 | 660 | 86 | 1088 | 39 | 316 | 542 | 897 |
| 16 | 1122 | 1241 | 542 | 732 | 26 | 1300 | 91 | 330 | 629 | 1050 |
| 17 | 745 | 789 | 340 | 327 | 10 | 677 | 49 | 422 | 299 | 770 |
| 18 | 778 | 859 | 156 | 557 | 220 | 933 | 8 | 169 | 645 | 822 |
| 19 | 678 | 892 | 367 | 481 | 17 | 865 | 44 | 185 | 386 | 615 |
| 20 | 252 | 274 | 223 | 83 | 11 | 317 | 56 | 112 | 68 | 236 |
